# Predicting for anti-(mutant) SARS-CoV-2 and anti-inflammation compounds of Lianhua Qingwen Capsules in treating COVID-19

**DOI:** 10.1186/s13020-022-00637-0

**Published:** 2022-07-07

**Authors:** Liang Hong, Min He, Shaoping Li, Jing Zhao

**Affiliations:** 1 State Key Laboratory of Quality Research in Chinese Medicine, Institute of Chinese Medical Sciences, University of Macau, Macau, China; 2 Department of Pharmaceutical Sciences, Faculty of Health Sciences, University of Macau, Macau, China; 3 Department of Pharmaceutical Engineering, School of Chemical Engineering, Xiangtan University, Xiangtan, China

**Keywords:** Lianhua Qingwen Capsules (LHQW), COVID-19, Molecular docking, Network pharmacology

## Abstract

**Background:**

Lianhua Qingwen Capsules (LHQW) is a traditional Chinese medicine prescription commonly used to treat viral influenza in China. There has been sufficient evidence that LHQW could effectively treat COVID-19. Nevertheless, the potential anti-(mutant) SARS-CoV-2 and anti-inflammation compounds in LHQW are still vague.

**Methods:**

The compounds of LHQW and targets were collected from TCMSP, TCMID, Shanghai Institute of Organic Chemistry of CAS database, and relevant literature. Autodock Vina was used to carry out molecular docking. The pkCSM platform to predict the relevant parameters of compound absorption in vivo. The protein–protein interaction (PPI) network was constructed by the STRING database. The Kyoto Encyclopedia of Genes and Genomes (KEGG) pathway enrichment analysis was carried out by Database for Annotation, Visualization, and Integrated Discovery (DAVID). The anti-(mutant) SARS-CoV-2 and anti-inflammation networks were constructed on the Cytoscape platform.

**Results:**

280 compounds, 16 targets related to SARS-CoV-2, and 54 targets related to cytokine storm were obtained by screening. The key pathways Toll-like receptor signaling, NOD-like receptor signal pathway, and Jak-STAT signaling pathway, and the core targets IL6 were obtained by PPI network and KEGG pathway enrichment analysis. The network analysis predicted and discussed the 16 main anti-SARS-CoV-2 active compounds and 12 main anti-inflammation active compounds. Ochnaflavone and Hypericin are potential anti-mutant virus compounds in LHQW.

**Conclusions:**

In summary, this study explored the potential anti-(mutant) SARS-CoV-2 and anti-inflammation compounds of LHQW against COVID-19, which can provide new ideas and valuable references for discovering active compounds in the treatment of COVID-19.

**Supplementary Information:**

The online version contains supplementary material available at 10.1186/s13020-022-00637-0.

## Background

Since the end of 2019, Severe Acute Respiratory Syndrome Coronavirus 2 (SARS-CoV-2) has caused an outbreak of Coronavirus Disease (COVID-19) in more than 200 countries/regions around the world. SARS-CoV-2 spreads faster, has a longer incubation time, and has more people infected than Severe Acute Respiratory Syndrome Coronavirus (SARS-CoV) and Middle East Respiratory Syndrome Coronavirus (MERS-CoV). The new coronavirus has become an urgent public health problem in the twenty-first century [1], and the emergence of virus mutants have strengthened the global spread of COVID-19 [2]. At present, Variants of concern (VOC) classified by the World Health Organization (WHO) include the alpha, beta, gamma, delta, and omicron strains, which exhibit high transmissibility, can reduce the neutralizing ability of antibodies, especially vaccine-elicited sera. The variants cause scientists to worry about the effectiveness of the vaccines [3–5]. So, it is crucial to find compounds that could be anti-mutant SARS-CoV-2.

The patients infected with SARS-CoV and MERS-CoV have symptoms of acute lung injury (ALI), systemic inflammatory response syndrome (SIRS), and acute respiratory distress syndrome (ARDS), and the patients infected with SARS-CoV-2 also have these symptoms. These clinical features are related to inflammation, which is caused by cytokines. The inflammatory plays an anti-virus effect when the coronavirus infects the host, but the inflammatory storm (also called cytokine storm) caused by the intense stimulation of the virus may cause more harm to the patient [6]. Therefore, effective suppression of cytokine storms is a key way to prevent the deterioration of COVID-19 patients. So far, there is no specific medicine for COVID-19. In China, a combination of Chinese and Western medicine is used to treat COVID-19 patients, and it has been clinically confirmed [7]. A retrospective cohort study has shown that Chinese herbal medicine can reduce mortality in patients with severe and critical Coronavirus disease 2019 [8]. Significantly, the National Health Commission recommended traditional Chinese medicine prescription LHQW as a therapeutic drug in the "New Coronavirus Pneumonia Diagnosis and Treatment Plan" (Trial Version 8) [9].

LHQW is derived from two classics prescriptions: Ma Xing Shi Gan Tang in the Han Dynasty and Yin Qiao San in the Qing Dynasty. LHQW comprises 11 Chinese herbal medicines, Gypsum Fibrosum (Shi Gao-SG) and Menthol (Bo He Nao-BHN). Among them, Chinese herbal medicines include Forsythiae Fructus (Lian Qiao-LQ), Lonicerae Japonicae Flos (Jin Yin Hua-JYH), Ephedrae Herba (Ma Huang-MH), Armeniacae Semen Amarum (Ku Xing Ren-KXR), Isatidis Radix (Ban Lan Gen-BLG), Dryopteridis Crassirhizomatis Rhizoma (Mian Ma Guan Zhong-MMGZ), Houttuyniae Herba (Yu Xing Cao-YXC), Pogostemonis Herba (Guang Huo Xiang-GHX), Rhei Radix et Rhizoma (Da Huang-DH), Rhodiolae Crenulatae Radix et Rhizoma (Hong Jing Tian-HJT) and Glycyrrhizae Radix et Rhizoma (Gan Cao-GC). The combined LHQW and western medicine treatment of COVID-19 can improve the clinical efficacy, significantly relieve the main symptoms and reduce the treatment time [10–14]. In vitro experiments had also confirmed that LHQW could inhibit the replication of SARS-CoV-2 in Vero E6 cells and significantly reduce the production of pro-inflammatory cytokines at the mRNA level [15]. Chen et al. used human exposure combined with ACE2 biochromatography to prove that rhein, forsythoside A, forsythoside I, neochlorogenic acid, and its isomers in LHQW have an inhibitory effect on the enzyme activity of ACE2 [16].

The wider recognition of LHQW is limited by its material basis research. Many cutting-edge technologies are valuable for research of material basis of Chinese medicine, such as chemometrics [17] and network pharmacology at present. Traditional Chinese medicine prescriptions use multiple compounds and multiple targets to exert their unique effects, consistent with the concept of network pharmacology. Through network pharmacology, drugs and disease targets can be analyzed from massive data, and their mechanisms can be understood. There have been 14 related articles on the network pharmacology about LHQW, and more relevant information can be found in Additional file 1: Table S7. Among them, there are 5 articles in English [18–22]. For example, Zheng et al. analyzed the entire target network that LHQW may act on and obtained key active compounds such as oleanolic acid, pseudoephedrine, luteolin, kaempferol, sitosterol, and quercetin from the functional modules [20]; Xia et al. obtained the core target Akt1 through network pharmacology and analyzed the potential binding mechanism of key active compounds (β-carotene, kaempferol, luteolin, naringenin, quercetin, Wogonin) in LHQW with Akt1 by molecular docking [21]. The strategies of these articles are debatable: firstly, the compounds collection of multiple articles is based on a single database, and the compounds data usually obtained by this approach is flawed. In addition, Zheng et al. collected the compounds of MMGZ from the TCMSP database, and the rest were collected from the Encyclopedia of Traditional Chinese Medicine database (ETCM) [20]. Due to the difference in compounds information in different databases, this method may cause defects to be superimposed and result in more significant errors than a single database ultimately. Secondly, the target collection comes from databases such as GeneCards. However, the content of the database is increasing over time, and as target data increases, more information about core targets will be obscured. Moreover, the research on targets is not sufficiently targeted. For example, virus-related targets research only targets ACE2 [18, 20]. However, more and more targets have been proved to play an essential role in the process of virus self-replication and invasion of host cells. These are all related to the accuracy and reliability of prediction results [23]. In addition, there are 9 LHQW related network pharmacology Chinese articles [24–32], which also include the problems above. For example, the compounds of 7 articles are collected only from the TCMSP database. Another problem is that they are all published in Chinese, making it hard for most scientists in the world to read them. Finally, the mutant and anti-mutant compounds are also worth researching with the emergence of SARS-CoV-2 mutants, but all 14 articles did not involve these.

**Fig. 1:**
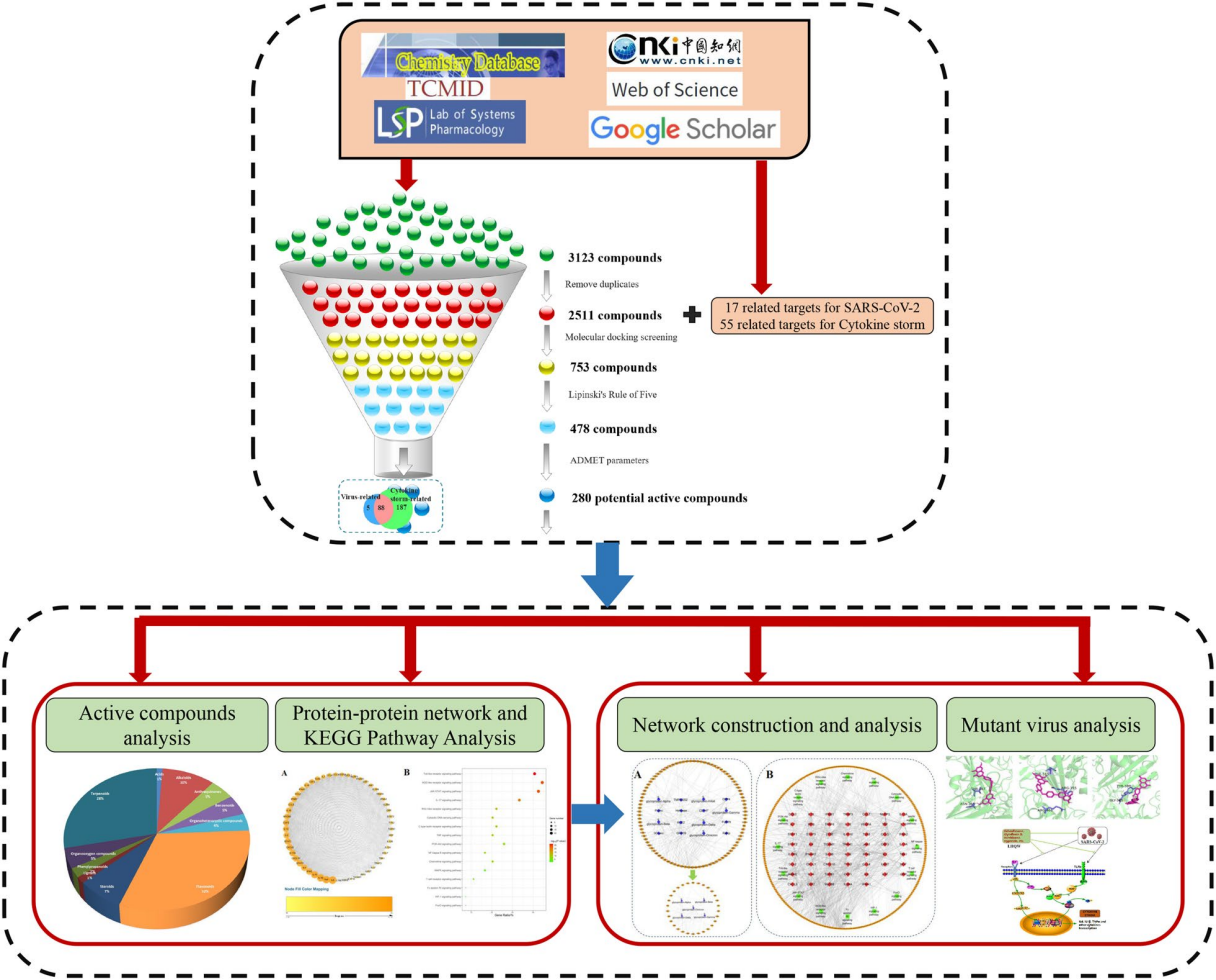
The flowchart of this study for predicting potential active compounds in LHQW against COVID-19

In this study, LHQW compounds database was constructed by integrating different databases and related literature. In terms of targets, the collection focused on anti-(mutant) SARS-CoV-2 and anti-inflammation and collected corresponding important targets from the literature. Finally, molecular docking, medicinal properties of compounds, and network pharmacology were adopted to obtain the potential anti-(mutant) SARS-CoV-2 and anti-inflammation compounds of LHQW in the treatment of COVID-19. The flowchart is illustrated in Fig. 1.

## Methods

### LHQW compounds database construction

The compounds of all Chinese herbal medicines were collected through the Traditional Chinese Medicine Systems Pharmacology database (TCMSP, http://tcmspw.com/tcmsp.php), Traditional Chinese Medicines Integrated Database (TCMID, http://119.3.41.228:8000/tcmid/), Shanghai Institute of Organic Chemistry of CAS (http://www.organchem.csdb.cn/) and related literature [33–62], and the 3d structure of all compounds were downloaded from Pubchem (https://pubchem.ncbi.nlm.nih.gov/). The compounds without structural information in Pubchem can be drawn by Chemdraw (Version 16.0, Cambridge Soft, USA) and saved as an SDF format file by ChemBio3D (Version 16.0, Cambridge Soft, USA), and finally used Open Babel GUI (Version 2.4.1) to batch convert SDF format files into mol2 format files. Subsequently, AutoDock Tools (Version1.5.6, Scripps Research, USA) saved the mol2 format file as a PDBQT coordinate file for molecular docking by Autodock Vina (Version1.1.2, Scripps Research, USA).

### Target database construction

Seventeen targets related to SARS-CoV-2 were obtained from the literature to analyze potential anti-(mutant) SARS-CoV-2 compounds in LHQW. Specifically, six structural proteins of SARS-CoV-2 were collected, including the original S glycoprotein [63], five variants of S glycoprotein (including the B.1.1.7 (Alpha) lineage, the B.1.351 (Beta) lineage, the B.1.1.28 (Gamma) lineage, the B.1.617.2 (Delta) lineage and the B.1.1.529 (Omicron) lineage) [2, 4], and Protein N [64]; four non-structural proteins of SARS-CoV-2 were collected, including papain-like protease(PLpro), 3C-like protease (3CLpro), Helicase [65], RNA polymerase(RdRp) [66]); and 6 targets that may bind to SARS-CoV-2, including Angiotensin-Converting Enzyme 2 (ACE2) [67], CD147 [68], Dipeptidyl Peptidase 4 (DPP4) [69], FURIN [70], Glucose Regulatory Protein 78 (GRP78) [71, 72] and transmembrane serine protease (TMPRSS2) [66]. There are related network pharmacology researches on the targets S glycoprotein, ACE2, PLpro, 3CLpro, RdRp, CD147, and DPP4 [73–77], but TMPRSS2, FURIN, Helicase, Protein N, and GRP78 have not yet been reported. In addition, 55 relevant targets of cytokine storm were collected from relevant literature [78–80] to explore the anti-inflammation effects of LHQW. Finally, the information on targets that belong to Homo sapiens was obtained from UniProt (https://www.uniprot.org/uniprot/), and the 3D structure of targets was downloaded from the PDB database (https://www.rcsb.org/).

### Molecular docking

Autodock Vina [81] was used for molecular docking. Before docking, the targets need to be dehydrated, added with polar hydrogen, and saved as a pdbqt format file. The docking box wrapped the potential bonding pocket as much as possible, and the parameter value of the configuration file (The config file) is exhaustiveness = 8, energy_range = 3, num_modes = 9. Information about cytokine-related and virus-related targets is shown in Additional file 1: Tables S1 and S2, respectively. After docking, the screening needs to meet two conditions: 1. The docking threshold needs to be less than − 7 kcal/mol, which has a better binding ability; 2. To avoid retaining too many compounds and find the better active compounds, the number of corresponding compounds for each target selection is less than or equal to 50 (satisfy condition 1).

### Analysis of the medicinal properties of LHQW compounds

The relevant parameters of compounds absorption in vivo were predicted using pkCSM (http://biosig.unimelb.edu.au/pkcsm/) [82]. The pkCSM is a user-friendly, freely available web interface. It could predict a range of absorption, distribution, metabolism, excretion, and toxicity (ADMET) properties for novel chemical entities based on the graph-based structural signature.

### PPI network and KEGG pathway enrichment analysis

With the background of "Homo sapiens", the protein–protein interaction (PPI) network of cytokine storm-related targets was constructed in the STRING database (https://string-db.org/). The Cytoscape 3.7.1 platform was used to visualize and analyze protein interaction networks.

The collected gene symbols of 55 cytokine storm-related targets were imported into DAVID 6.8 (https://david.ncifcrf.gov/) [83]. Identifier, Background, and Pathways menus were selected as OFFICIAL_GENE_SYMBOL, Homo sapiens, KEGG_PATHWAY, and KEGG pathway enrichment analysis results can be obtained.

### Network construction

According to the above results. The anti-(mutant) SARS-CoV-2/anti-inflammation network can be constructed separately by importing the Cytoscape 3.7.1 platform to study the potential anti-SARS-CoV-2 and anti-inflammation effects of LHQW.

## Results and discussion

### Compounds collection and screening

A total of 3123 compounds of the herbal medicines contained in LHQW were collected from the databases and related literature. There are 612 repetitive compounds, and 2511 compounds were docked with related targets using Autodock Vina. The binding energy of the docking results can be used to evaluate the degree of binding between the ligand and the receptor. The ligand and receptor molecule conformation can be stably combined when affinity is less than 0-the greater the absolute affinity value, the stronger the stability. After docking, 753 chemical compounds can be screened out by the docking threshold setting under '2.3'.

After that, the recognized Lipinski's Rule of Five can be used to screen [84]. The rule states that if Octanol–water partition coefficient (logp) ≤ 5, Molecular Mass (MW) ≤ 500, hydrogen bond acceptor (HBA) ≤ 10, and Hydrogen bond donor (HBD) ≤ 5, the compound is more likely to have membrane permeability and is easily absorbed by passive diffusion in the human intestine. If the molecule violates two or more of these rules, it is unlikely to reach the blood from the intestine. These parameters are critical in the drug development process because they determine the fate of the molecule in vivo. According to Lipinski's Rule of Five, compounds that violate two or more rules are deleted. These compounds are considered less likely to have good oral availability, whereby 478 compounds were screened.

**Fig. 2:**
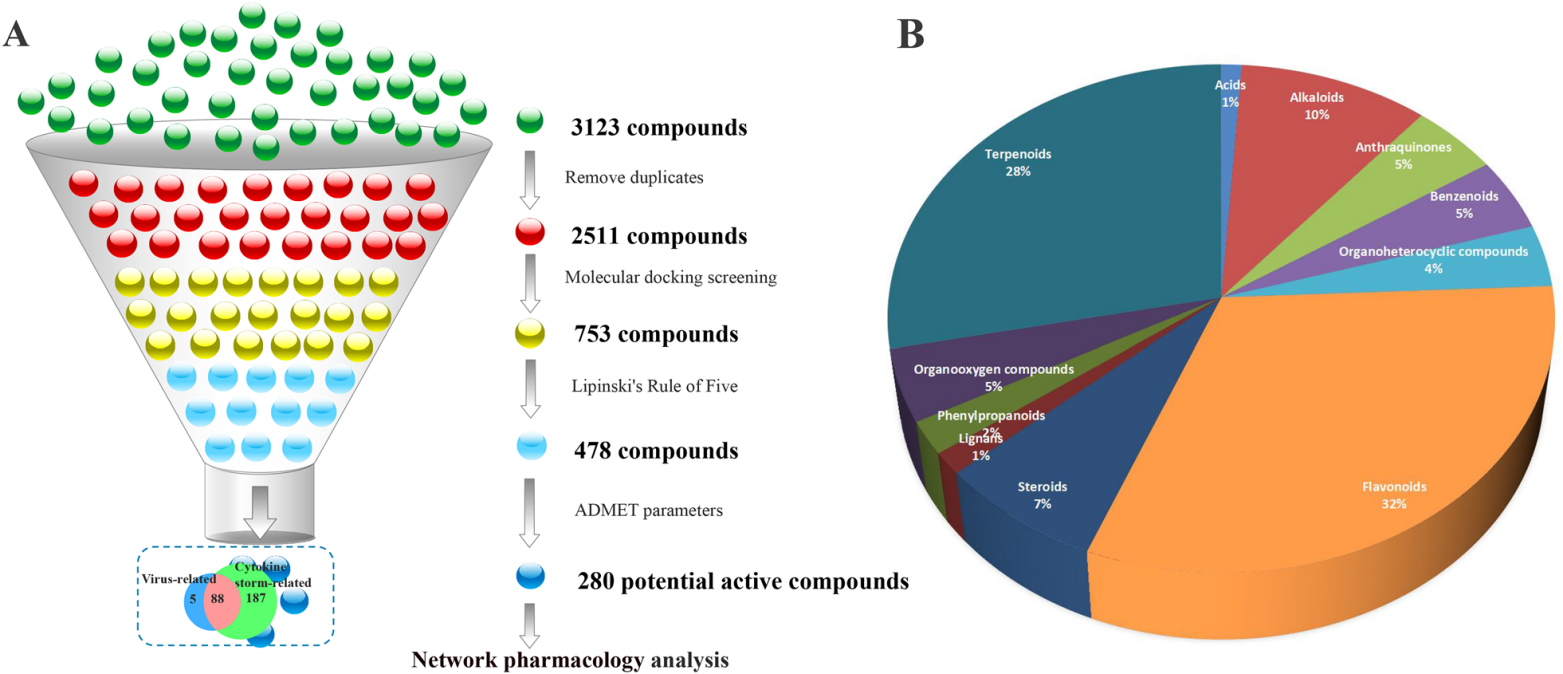
**A** The Compounds screening process, methods include Remove duplicates, Molecular docking screening, Lipinski's Rule of Five, and ADMET parameters. **B** Distribution of potentially active compounds, mainly Flavonoids, Terpenoids, and Alkaloids

The compounds screened by using Lipinski's Rule of Five need to have good pharmacokinetic characteristics to have potential therapeutic effects. Among the ADMET parameters predicted in the pkCSM server, two important in vivo absorption parameters were further selected to screen compounds, that is, Caco2 permeability > 0.9, the compound is considered to have a higher Caco2 permeability; Intestinal absorption (human) > 30%, the compound can be considered to be well absorbed in the human intestine. However, Lipinski's Rule of Five and ADMET parameters are not a panacea. Many compounds violate these rules with clear pharmacological activities [85]. It was found that 13 herbal medicines contained some active compounds through searching the literature, which were deleted by the system if they did not meet the screening conditions. There were 46 compounds considering the potential significance of these compounds that will not be deleted by the next two parameters after meeting the threshold screening of molecular docking. According to two parameters in ADMET, 280 active compounds can be screened. Finally, the docking results are shown in Additional file 1: Table S3, the medicinal properties of each compound are shown in Additional file 1: Table S4, and the threshold range of each target is shown in Additional file 1: Table S5. The compounds screening process is shown in Fig. 2A.

### Analysis of potentially active compounds

After screening, 280 potentially active compounds and 70 targets (16 virus-related targets and 54 cytokine storm-related targets) can be obtained. Each target corresponds to an average of 12.64 compounds, and each compound corresponds to an average of 3.16 targets. It shows that different active compounds can act on the same target, and the same active compound can also act on different targets, reflecting the multi-compounds and multi-targets characteristics of Chinese medicine. The 280 potentially active compounds were classified, as shown in Fig. 2B and Additional file 1: Table S6. Flavonoids, Terpenoids, and Alkaloids accounted for 70% of the total. In addition, there are Steroids, Organooxygen compounds, Phenylpropanoids, Anthraquinones, Organoheterocyclics compounds, etc., which indicates that there are many classes of compounds that could play a potentially active role. Flavonoids [86, 87], terpenoids [88, 89], and alkaloids [90, 91], which account for a relatively large proportion, have been reported in the literature to have anti-virus and anti-inflammation effects. Among them, the number of active compounds corresponding to each herbal medicine is: BLG (n = 26), DH (n = 24), GC (n = 87), GHX (n = 21), LQ (n = 45), JYH (n = 31), KXR (n = 15), HJT (n = 7), MH (n = 38), MMGZ (n = 17), YXC (n = 52). According to the compatibility principle of the traditional Chinese medicine prescription "Monarch Minister Assistant and Guide" [18], LQ and JYH are the Monarch medicines. They are the therapeutic drugs for the main symptoms and the two synergistic effects; both have the effects of clearing heat, detoxifying, and dispelling wind-heat; MH, SG, and KXR are Minister medicines. MH can help to relieve the lung and relieve asthma, relieve the surface, SG clears internal heat, KXR relieves cough and relieve asthma; BLG, MMGZ, YXC, BHN, GHX, DH, HJT are Assistant medicines, which can clear the lungs and detoxify, relieve the lungs and heat, reduce dampness and turbidity, and regulate qi and neutralize; GC as ‘Guide medicine’ can coordinate all medicines. These indicate that LHQW has anti-viral and anti-inflammatory effects.

### PPI network and KEGG pathway enrichment analysis of cytokine storm-related targets

**Fig. 3:**
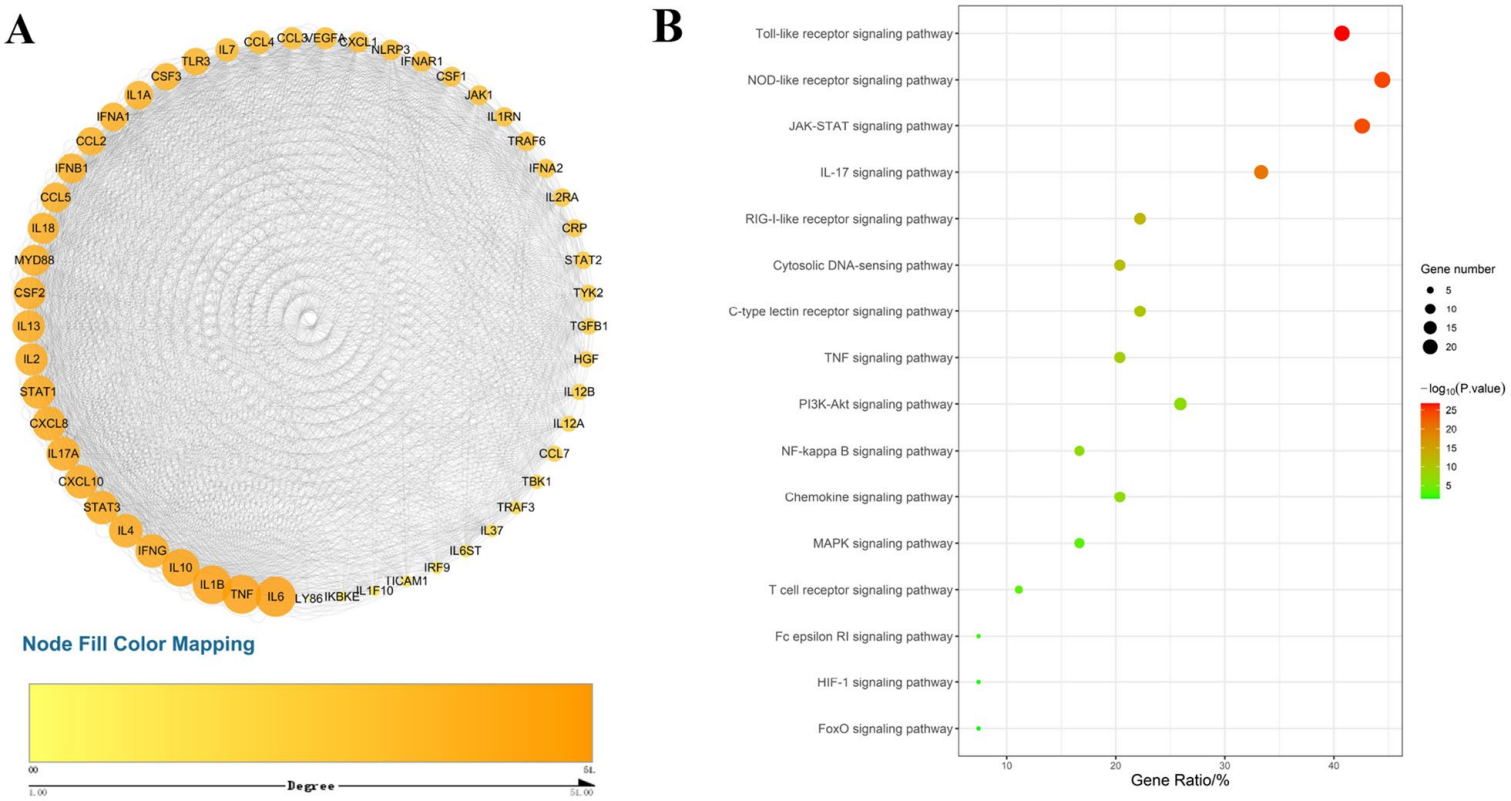
**A** The protein–protein interaction (PPI) network. In the network, the relationship between protein and protein is closely connected. Node Fill Color Mapping refers to the color of each target point in the PPI network from yellow to orange, representing the degree value of the target point from small to large. **B** KEGG enrichment analysis of cytokine targets. The bubble color is green to red, representing that the P value of the pathway is from large to small. Gene number refers to the number of all annotated genes contained in the pathway. The larger the bubble, the greater the number of all annotated genes in the pathway. Gene Ratio refers to the ratio of the number of genes located in the pathway among all annotated genes to the number of all annotated genes

After molecular docking, 54 cytokine storm-related targets were screened to construct a PPI network, including 53 nodes and 1876 edges (Fig. 3A). The PPI network is characterized based on the degree value. The greater the degree, the larger the node, the darker the color, and the higher the importance. Each edge represents the interaction between proteins. The average degree of the network is 35.4, and there are 30 targets more than this value, indicating that the network targets are closely connected. There are 23 targets with a degree value greater than 40, as shown in Table 1.

**Table 1:** The targets information with a degree value greater than 40 in the PPI network

Gene name	Uniprot ID	Degree	Gene name	Uniprot ID	Degree
Interleukin 6 (IL6)	P05231	51	Interleukin 13 (IL13)	P35225	45
Tumor necrosis factor (TNF)	P01375	50	Granulocyte–macrophage colony-stimulating factor (CSF2)	P04141	45
Interleukin-1 beta (IL1B)	P01584	50	Myeloid differentiation primary response protein (MYD88)	Q99836	44
Interleukin 10 (IL10)	P22301	49	Interleukin 18 (IL18)	Q14116	44
Signal transducer and activator of transcription 1 (STAT1)	P42224	46	Interferon beta (IFNB1)	P01574	43
Interferon gamma (IFNG)	P01579	46	C–C motif chemokine 5 (CCL5)	P13501	43
Interleukin 17A (IL17A)	Q16552	46	C–C motif chemokine 2 (CCL2)	P13500	42
Signal transducer and activator of transcription 3 (STAT3)	P40763	46	Interferon alpha-1 (IFNA1)	P01562	42
Interleukin 4 (IL4)	P05112	46	Interleukin-1 alpha (IL1A)	P01583	42
C-X-C Motif Chemokine Ligand 10 (CXCL10)	P02778	46	Granulocyte colony-stimulating factor (CSF3)	P09919	41
C-X-C Motif Chemokine Ligand (CXCL8)	Q14116	46	Toll-like receptor 3 (TLR3)	O15455	41
Interleukin 2 (IL2)	P60568	45			

The information of 54 cytokines storm-related targets was imported into the DAVID database for KEGG pathway enrichment analysis. Then, 16 signal pathways (P < 0.05) caused by COVID-19 were visualized, as shown in Fig. 3B. It can be seen that the Toll-like signal pathway, the NOD-like receptor signal pathway, and the Jak-STAT signal pathway have the largest number of targets and the smallest P.value, which are the key signal pathways. They contain IL6 with a degree value greater than 50 in the PPI network. IL6 has the highest degree value and an interactive relationship with 51 targets. It means IL6 plays an important role in the entire cytokine storm network.

As shown in Fig. 6, when SARS-CoV-2 invades the host, immune cells detect pathogen-related molecular patterns (PAMPs) from the virus through the pattern recognition receptors (PRRs) on the cell membrane, a typical one of which is Toll-like receptors (TLRs). Viral genomic RNA or intermediate products will be recognized by endosomal RNA receptors TLR7 and TLR8 in the process of viral replication, thereby activating downstream signaling pathways, and leading to the secretion of cytokines (including IL6, etc.) [92]. SARS-CoV-2 genome can also activate the nucleotide-binding oligomerization domain (NOD)-like receptors (NLRs) inflammasome and cytokine release in macrophages [93]. The cytokines bind with the corresponding receptors and will further activate the Jak-STAT signaling pathway, PI3K/AKT pathway [94], and NF-κB signaling pathway, thereby promoting the expression of nuclear DNA to generate a large number of cytokines, and inducing the continuous expansion of cytokines [95]. The Jak-STAT signaling pathway is a common pathway for the signal transduction of many cytokines and is widely involved in cell proliferation, differentiation, apoptosis, and inflammation, which can activate downstream signaling pathways to coordinate cell responses through targets gene expression [96]. They all associate with IL6, indicating IL6 plays a more important role in the PPI network. IL6 has the highest degree value in the network, which is one of the core cytokines in the host's response to SARS-CoV-2 infection. Due to the strong correlation between IL6 and COVID-19, studies have considered IL-6 levels in serum as potential diagnostic markers, disease severity stratification indicators, and prognostic indicators. The researchers are exploring targeting monoclonal antibodies against IL6 and its receptor, such as tocilizumab (Actemra) [97]. IL6 as the core target can provide a simpler design idea for subsequent verification.

### Network analysis

In this section, the compounds and targets screened through molecular docking and medicinal properties were used to construct anti-(mutant) SARS-CoV-2 and anti-inflammation networks, respectively, to explore the potential mechanism of LHQW in the treatment of COVID-19.

**Fig. 4:**
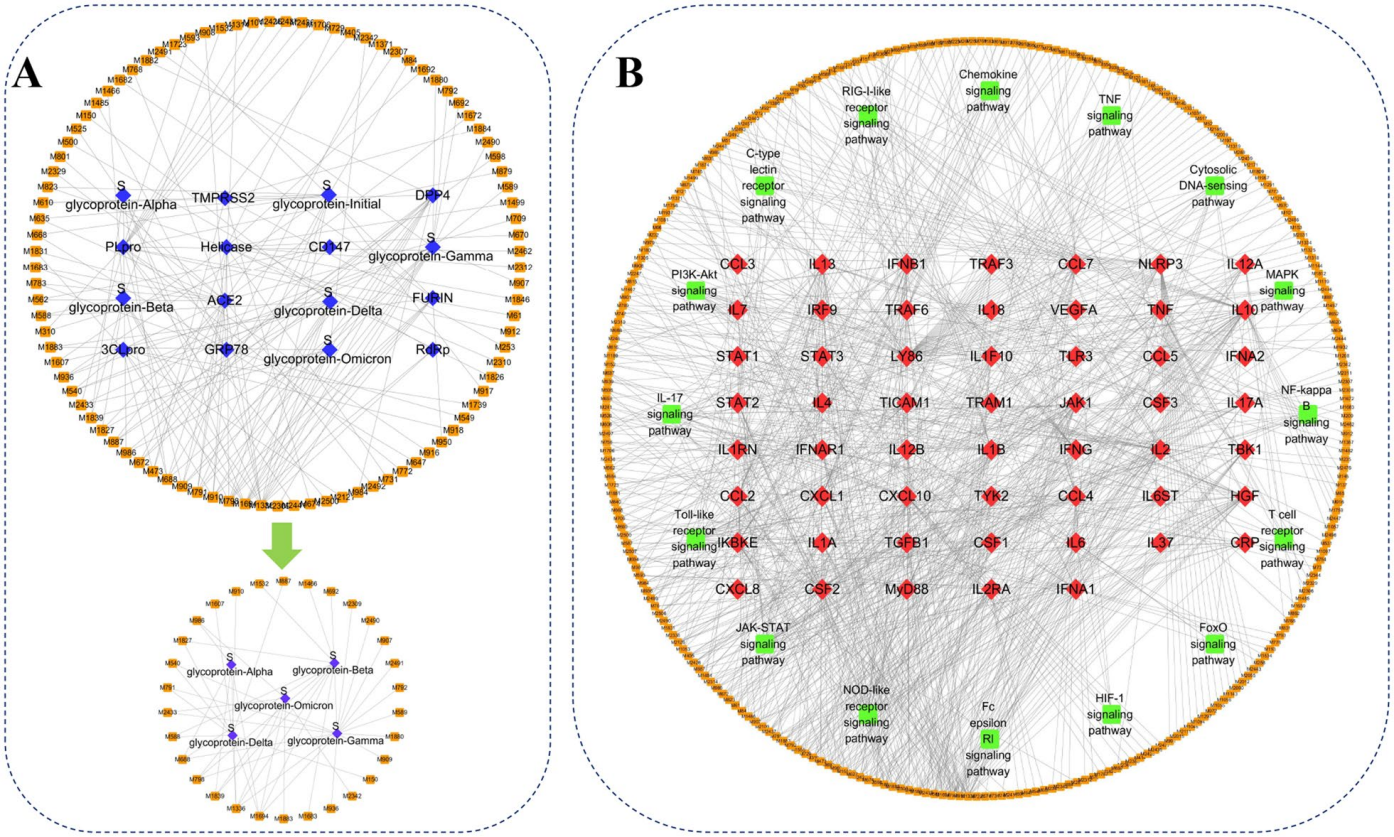
**A** Anti-(mutant) SARS-CoV-2 and **B** anti-inflammation network. The black connection indicated that there is a connection between nodes. Orange rectangle = active compounds of LHQW; blue diamond = targets related to SARS-CoV-2; red diamond = targets related to cytokine storm; green square = KEGG pathway

As shown in Fig. 4A, an anti-SARS-CoV-2 compounds-targets network was constructed by 93 potentially active compounds and 16 virus-related targets. In this network, the degree value of 16 compounds is greater than or equal to 3, as shown in Table 2. Among the selected compounds, Hypericin has been reported to have the activity of inhibiting SARS-CoV-2 in vitro [98]; Hispaglabridin B showed activity against influenza virus [99]; (−)-Epicatechin-3-O-gallate can inhibit the in vitro activity of chronic hepatitis C virus [100]; Glycyrrhizin can protect cells from influenza A virus infection [101]. However, the anti-SARS-CoV-2 effects of these compounds need to be further confirmed. The remaining compounds, Ochnaflavone, Glyinflanin D and Xambioona, etc. have not yet been reported for their anti-viral effects. ACE2 can interact with SARS-CoV-2 surface ligands and is a core target that has been proven to mediate virus invasion into host cells [102]. Of these compounds, 9 are related to ACE2, indicating that LHQW is likely related to blocking the binding of the virus to ACE2 when treating COVID-19.

**Table 2:** The potential anti-(mutant) SARS-CoV-2 active compounds

Mol ID	Compounds	Degree	Sources	Classifications
M1336	Ochnaflavone	12	JYH	Flavonoids
M1694	Hypericin	9	LQ	Flavonoids
M798	Xambioona	7	GC	Flavonoids
M910	Glyinflanin D	6	GC	Flavonoids
M688	Hispaglabridin B	5	GC	Flavonoids
M791	Kanzonol E	5	GC	Flavonoids
M909	Glyinflanin C	5	GC	Flavonoids
M1827	Taraxasterol acetate	3	LQ	Terpenoids
M1839	3β-Acetoxy-11-en-olean-28,13-olide	3	LQ	Terpenoids
M2433	Cepharadione A	3	YXC	Alkaloids
M473	(−)-Epicatechin-3-O-gallate	3	DH	Flavonoids
M540	Glycyrrhizin	3	DH, GC	Terpenoids
M672	Licorice-saponin F3_qt	3	GC	Terpenoids
M887	Deoxyglabrolide	3	GC	Terpenoids
M936	Licoflavone B	3	GC	Flavonoids
M986	Friedelin	3	GHX	Terpenoids

**Fig. 5:**
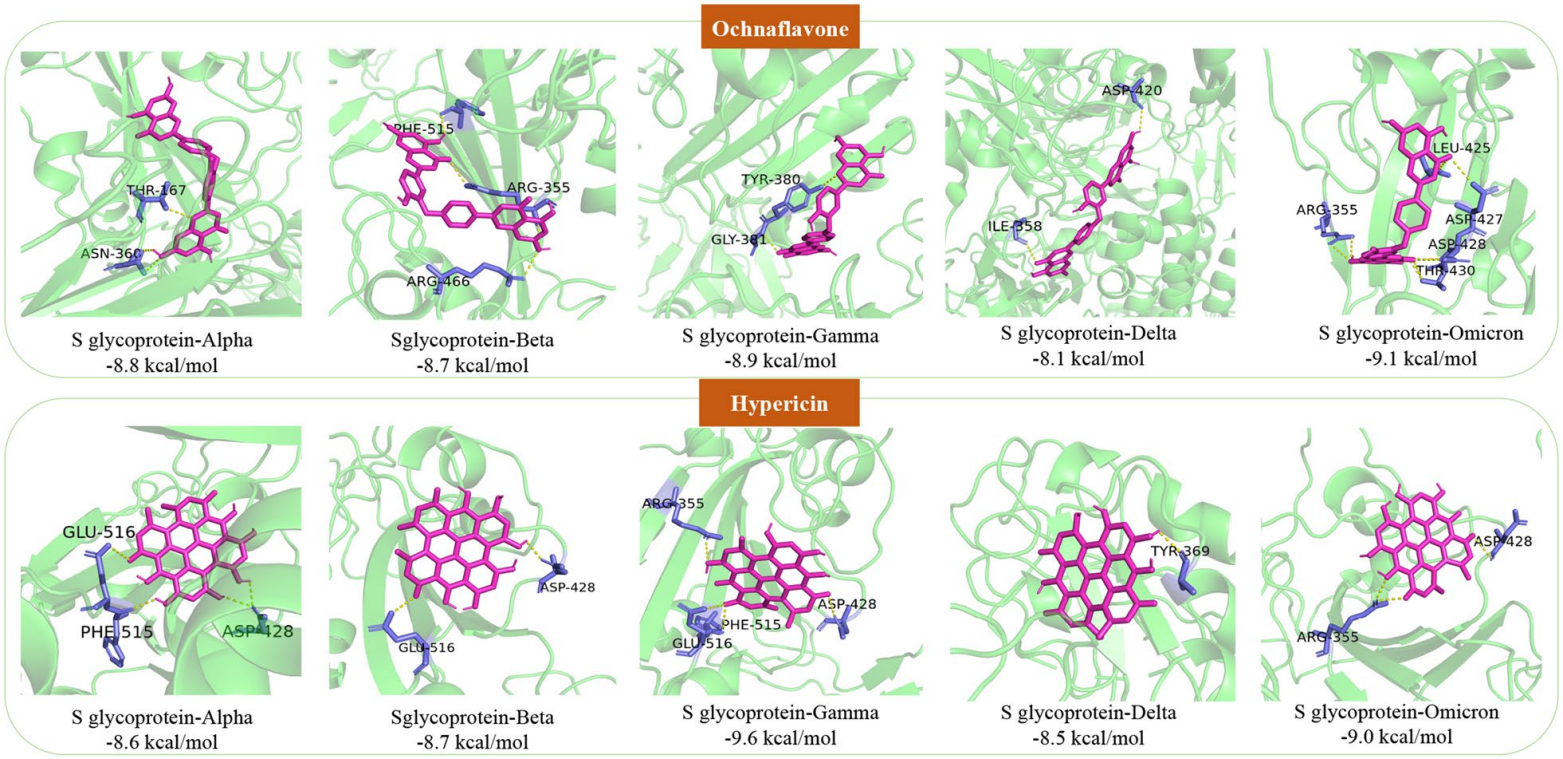
The predicted binding mode of Ochnaflavone and Hypericin with five mutant S glycoproteins. The light magenta color represents the active compounds. Translucent green is the mutant S glycoproteins; Slate is the amino acid residues

Since the mutation of SARS-CoV-2 greatly enhanced the spread of COVID-19, we also collected five significant variants to predict anti-mutant virus compounds in LHQW. The further anti-mutant virus network was constructed by 30 potentially active compounds and 5 mutant virus targets. In this network, only 2 compounds (−)-Epicatechin-3-O-gallate and Licorice-saponin F3_qt were not present in the 16 compounds screened by the anti-SARS-CoV-2 network in the previous step. Ochnaflavone and Hypericin are associated with the five mutant viruses, indicating that these two compounds are potentially active compounds against the mutant viruses in LHQW. Ochnaflavone and Hypericin are derived from JYH and LQ, respectively, and JYH and LQ are monarch medicines (the most important medicines) in LHQW formula, which further shows that these compounds are worthy of study. The potential binding of Ochnaflavone and Hypericin with the five mutant S glycoproteins is shown in Fig. 5. It is predicted that Ochnaflavone has a hydrogen bond interaction with THR-167, ASN-360 of S glycoprotein-Alpha, PHE-515, ARG355, ARG-466 of S glycoprotein-Beta, TYR-380, GLY-381 of S glycoprotein-Gamma, ASP-420, ILE-358 of S glycoprotein-Delta, and ARG-355, LEU-425, ASP-427, ASP-428, THR-430 of S glycoprotein-Omicron, respectively. It is predicted that Hypericin has a hydrogen bond interaction with GLU-516, PHE-515, ASP-128 of S glycoprotein-Alpha, ASP-428, GLY-516 of S glycoprotein-Beta, ARG-355, GLU-516, PHE-515, ASP-428 of S glycoprotein-Gamma, TYR-369 of S glycoprotein-Delta, and ARG-355, ASP-428 of S glycoprotein-Omicron, respectively. Ochnaflavone and Hypericin bind tightly to five targets through multiple hydrogen bonds. Therefore, they are likely to be potential active compounds against mutant viruses in LHQW and worthy of further analysis.

As shown in Fig. 4B, the compounds-targets-pathways anti-inflammation network was constructed by 275 potential compounds, 54 cytokine-related targets, and 16 pathways. In this network, there are 12 compounds whose degree value is greater than or equal to 9, as shown in Table 3. Among them, Ochnaflavone can exert anti-inflammation effects by inhibiting NF-κB [103]; In macrophages, Hypericin suppressed LPS induced NO production with a concomitant decrease in the levels of pro-inflammatory cytokines [104]; (−)-Bicuculline and Rutaecarpine reduce inflammation by inhibiting the production of pro-inflammatory cytokines [105, 106]; Suspensine A has proven anti-inflammatory activity [107]. However, seven compounds, Glyinflanin D, Xambioona, Cepharadione A, etc., have not yet been reported for their anti-inflammation effects. All 12 compounds could act on the Toll-like signaling pathway, the NOD-like receptor signal pathway, and the Jak-STAT signaling pathway through corresponding targets; Ochnaflavone, Xambioona, and Glyinflanin D could act on three key pathways by IL6, which may be an important way for LHQW to exert its anti-inflammation effects.

**Table 3:** The potential anti-inflammation active compounds

Mol ID	Compounds	Degree	Sources	Classifications
M1336	Ochnaflavone	21	JYH	Flavonoids
M910	Glyinflanin D	19	GC	Flavonoids
M798	Xambioona	18	GC	Flavonoids
M1694	Hypericin	11	LQ	Flavonoids
M589	(2S)-2-[4-hydroxy-3-(3-methylbut-2-enyl)phenyl]-8,8-dimethyl-2,3-dihydropyrano[2,3-f]chromen-4-one	11	GC	Flavonoids
M1884	Rutaecarpine	10	LQ	Alkaloids
M2433	Cepharadione A	10	YXC	Alkaloids
M1880	Suspensine A	9	LQ	Alkaloids
M1883	(−)-Bicuculline	9	LQ	Alkaloids
M2498	Strigone	9	YXC	Terpenoids
M2501	5-Deoxystrigol	9	YXC	Terpenoids
M598	(E)-1-(2,4-dihydroxyphenyl)-3-(2,2-dimethylchromen-6-yl)prop-2-en-1-one	9	GC	Flavonoids

**Fig. 6:**
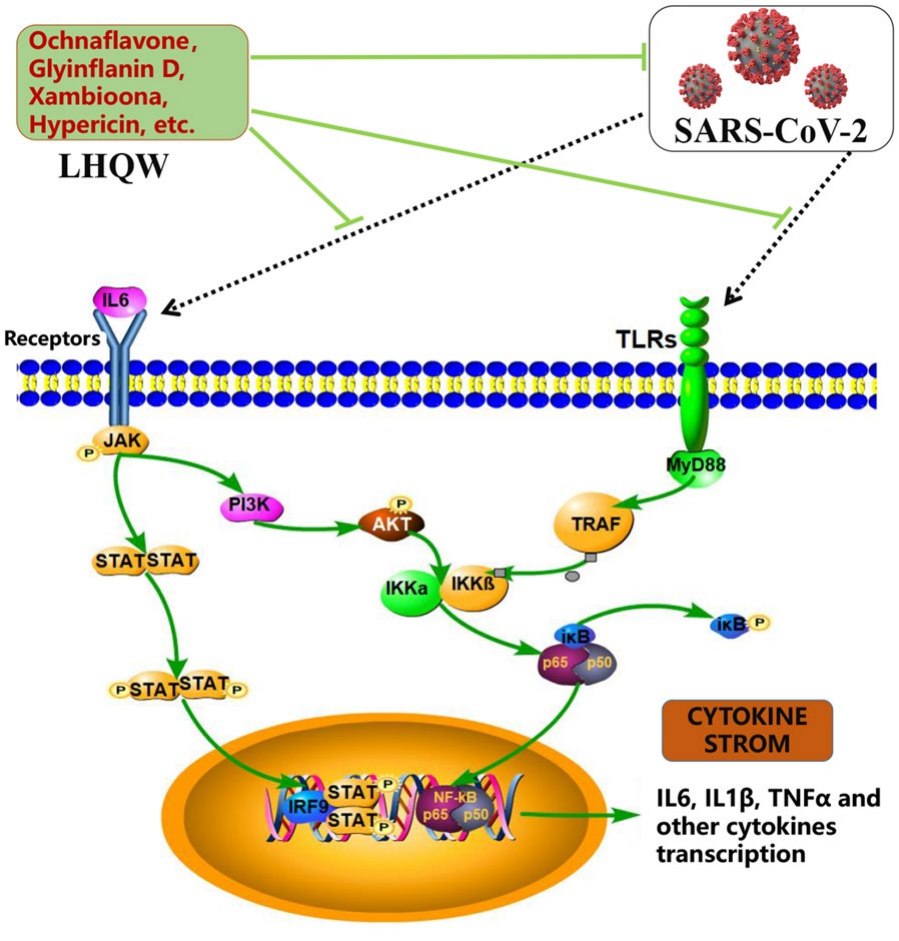
The underlying mechanism of LHQW in the treatment of COVID-19. The active compounds in LHQW can treat COVID-19 in two ways: anti-(mutant) SARS-CoV-2 and anti-inflammation

As shown in Fig. 6, LHQW can play a role in anti-(mutant) SARS-CoV-2 and anti-inflammation through the potential active compounds screened, Ochnaflavone, Glyinflanin D, Hypericin, Xambioona, etc. However, it is necessary to use this information to carry out relevant experiments in the follow-up to explain these mechanisms more convincingly.

The more important active compounds were obtained from the anti-(mutant) SARS-CoV-2 and anti-inflammation network by analyzing the degree value. We chose the degree value of 3 in the anti-SARS-CoV-2 network and the degree value of 9 in the anti-inflammation network as the threshold to obtain and analyze the most critical active compounds, and 24 active compounds were screened. However, this method is largely controversial. There is no standard for threshold setting from the current network analysis, and many methods are adopted, such as selecting the top 3 [108] or top 10 [109, 110] of the degree value directly; Or combining topological parameters such as degree, Betweenness Centrality, and Closeness Centrality [111, 112]. In addition, it was found that only Glycyrrhizin has been confirmed to exist in LHQW through literature mining [113–116], which shows that the screening results need further compound verification.

## Conclusion

This study used molecular docking, medicinal properties of compounds, and network pharmacology to explore the anti-(mutant) SARS-CoV-2 and anti-inflammation compounds from LHQW in treating COVID-19. Firstly, the potential active compounds in LHQW were obtained from molecular docking and medicinal properties of compounds; the PPI network and KEGG pathway were analyzed from the perspective of cytokine storm, and it was found that each target was closely connected, and three key pathways and one core target were obtained. Finally, the key active compounds were obtained from the network analysis. In summary, all works showed that the active compounds in LHQW could inhibit or activate the targets, thereby producing anti-(mutant) SARS-CoV-2 effects or regulating signal pathways to exert anti-inflammation effects. This research can provide some valuable clues for the wider application of LHQW in aginst COVID-19 and the development of new drug strategies to control SARS-CoV-2.

## Supplementary Information

**Additional file 1: Table S1.** List of related targets for cytokine storm. **Table S2.** List of related targets for SARS-CoV-2. **Table S3.** Results of docking studies. **Table S4.** Medicinal properties of LHQW active compounds. **Table S5.** Results of docking studies-Affinity range of the compounds corresponding to each target. **Table S6.** Compounds database of LHQW. **Table S7.** Information about 14 articles related to LHQW Network Pharmacology.

## Data Availability

The datasets used and/or analyzed during the current study are available from the corresponding author on reasonable request.

## Abbreviations

COVID-19: Corona Virus Disease 2019
LHQW: Lianhua Qingwen Capsules
TCMSP: Traditional Chinese Medicine systems pharmacology
KEGG: Kyoto Encyclopedia of Genes and Genome
DAVID: Database for Annotation, Visualization and Integrated Discovery
PPI: Protein–protein interaction
IL-6: Interleukin- 6
3CLpro: 3C-like protease
ACE2: Angiotensin-Converting Enzyme 2
DPP4: Dipeptidyl Peptidase 4
GRP78: Glucose Regulatory Protein 78
TMPRSS2: Transmembrane protease serine 2
RdRp: RNA polymerase
PLpro: Papain-like protease
SARS-CoV-2: Severe Acute Respiratory Syndrome Coronavirus 2
IFNB: Interferon beta
