# Supplementary material for: Predicting for anti-(mutant) SARS-CoV-2 and anti-inflammation compounds of Lianhua Qingwen Capsules in treating COVID-19
Source: Chin Med. 2022 Jul 7;17:84. doi: 10.1186/s13020-022-00637-0 (PMC9261255; doi:10.1186/s13020-022-00637-0)
Supplement: Supplementary file 1 — Additional file 1: Table S1. List of related targets for cytokine storm. Table S2. List of related targets for SARS-CoV-2. Table S3. Results of docking studies. Table S4. Medicinal properties of LHQW active compounds. Table S5. Results of docking studies-Affinity range of the compounds corresponding to each target. Table S6. Compounds database of LHQW. Table S7. Information about 14 articles related to LHQW Network Pharmacology. [file 13020_2022_637_MOESM1_ESM.docx]

Table S1 List of related targets for cytokine storm

| **Target protein** | **Gene name** | **Uniprot ID** | **PDBID** | **X** | **Y** | **Z** | **Vina_Box** |
| --- | --- | --- | --- | --- | --- | --- | --- |
| Interleukin 2 | IL2 | P60568 | 1z92 | 3.487 | -35.421 | 3.913 | 18×18×24 |
| Interleukin 7 | IL7 | P13232 | 3di3 | 26.877 | 41.622 | 51.420 | 18×20×22 |
| C-C motif chemokine 3 | CCL3 | P10147 | 5d65 | 40.215 | 99.629 | 49.641 | 18×16×22 |
| Interleukin 1 Beta | IL1B | P01584 | 4I1B | 7.839 | 11.497 | -7.880 | 24×20×18 |
| C-Reactive Protein | CRP | P02741 | 1lj7 | 133.673 | 81.779 | 175.070 | 20×20×22 |
| Interleukin 6 | IL6 | P05231 | 1p9m | -43.249 | 172.995 | 45.212 | 14×14×14 |
| Interleukin-12 subunit beta | IL12B | P29460 | 3hmx | 35.584 | 18.567 | 35.427 | 24×24×22 |
| Interleukin-12 subunit alpha | IL12A | P29459 | 3hmx | 35.958 | 44.026 | 66.233 | 16×24×30 |
| Tumor Necrosis Factor | TNF | P01375 | 2az5 | -19.568 | 74.641 | 34.759 | 14×14×12 |
| Interferon gamma | IFNG | P01579 | 1fg9 | -11.384 | 0.778 | 7.455 | 12×12×12 |
| Interleukin 4 | IL4 | P05112 | 3bpn | 8.795 | -17.281 | 14.123 | 18×18×18 |
| Interleukin 10 | IL10 | P22301 | 1j7v | 12.036 | 18.569 | 18.569 | 12×12×12 |
| Interleukin 13 | IL13 | P35225 | 3bpo | -8.549 | -6.120 | -21.032 | 14×14×14 |
| Transforming growth factor beta-1 proprotein | TGFB1 | P01137 | 4kv5 | 12.154 | 26.080 | 13.549 | 20×20×20 |
| Toll-like receptor 3 | TLR3 | O15455 | 3ulv | -55.519 | 1.511 | -19.336 | 14×14×14 |
| Lymphocyte antigen 86 | LY86 | O95711 | 3b2d | 23.893 | -29.337 | -9.149 | 12×12×12 |
| Myeloid differentiation primary response protein | MyD88 | Q99836 | 3mop | 74.446 | 77.768 | 103.382 | 20×20×20 |
| Translocating chain-associated membrane protein 1 | TRAM1 | Q15629 | SWISS-MODEL | 16.609 | 45.743 | 15.567 | 16×16×16 |
| TIR domain-containing adapter molecule 1 | TICAM1 | Q8IUC6 | 4c0m | 6.718 | 60.799 | 44.408 | 14×14×14 |
| Serine/threonine-protein kinase TBK1 | TBK1 | Q9UHD2 | 4im2 | 107.827 | 12.268 | -28.978 | 20×20×20 |
| Inhibitor of nuclear factor kappa-B kinase subunit epsilon | IKBKE | Q14164 | SWISS-MODEL | 94.191 | 16.978 | -12.089 | 14×14×14 |
| TNF Receptor Associated Factor 6 | TRAF6 | Q9Y4K3 | 3hcu | -27.894 | 24.649 | -60.100 | 18×20×20 |
| Interleukin-6 receptor subunit beta | IL6ST | P40189 | 1p9m | -87.759 | 190.202 | 49.947 | 20×20×20 |
| Interleukin-8 | IL8 | P10145 | 1qe6 | 35.305 | 11.886 | -6.304 | 18×20×18 |
| C-C Motif Chemokine Ligand 5 | CCL5 | P13501 | 5dnf | -5.642 | -13.265 | 31.601 | 18×20×14 |
| C-C motif chemokine 2 | CCL2 | P13500 | 2bdn | 39.614 | 7.225 | 98.189 | 20×18×14 |
| C-X-C Motif Chemokine Ligand 10 | CXCL10 | P02778 | 1o7y | 48.308 | -2.846 | 18.921 | 20×18×18 |
| Interleukin 18 | IL18 | Q14116 | 3wo4 | -25.154 | -4.099 | 0.841 | 20×18×18 |

Table S1 List of related targets for cytokine storm

| **Target protein** | **Gene name** | **Uniprot ID** | **PDBID** | **X** | **Y** | **Z** | **Vina_Box** |
| --- | --- | --- | --- | --- | --- | --- | --- |
| NACHT, LRR and PYD domains-containing protein 3 | NLRP3 | Q96P20 | 2naq | -12.848 | -1.090 | -1.665 | 20×18×18 |
| Interferon Alpha 1 | IFNA1 | P01562 | 3ux9 | 24.322 | 73.923 | 27.817 | 20×20×20 |
| Interferon Alpha 2 | IFNA2 | P01563 | 3se3 | 40.525 | -42.092 | -10.811 | 20×20×20 |
| Interferon beta | IFNB1 | P01574 | 1au1 | 4.106 | 13.472 | 31.693 | 20×20×20 |
| Interferon alpha/beta receptor 1 | IFNAR1 | P17181 | 3se3 | 27.549 | -18.944 | 15.995 | 14×14×14 |
| Tyrosine-protein kinase JAK1 | JAK1 | P23458 | 6C7Y | -10.698 | 30.200 | -4.723 | 20×20×20 |
| Non-receptor tyrosine-protein kinase TYK2 | TYK2 | P29597 | 3LXN | -6.996 | 4.349 | 17.924 | 20×20×20 |
| Signal transducer and activator of transcription 1-alpha/beta | STAT1 | P42224 | 1yvl | -28.774 | 1.100 | 116.979 | 20×20×20 |
| Signal transducer and activator of transcription 2 | STAT2 | P52630 | 6UX2 | -26.723 | 8.979 | -2.388 | 14×14×14 |
| Interferon regulatory factor 9 | IRF9 | Q00978 | SWISS-MODEL | 35.185 | 26.341 | 14.438 | 16×16×16 |
| TNF Receptor Associated Factor 3 | TRAF3 | Q13114 | 1KZZ | -14.475 | 46.181 | 11.878 | 16×16×16 |
| Interleukin-1 alpha | IL1A | P01583 | 5uc6 | 96.119 | 64.950 | 111.994 | 16×16×16 |
| Interleukin-17A | IL17A | Q16552 | 4hsa | -55.134 | 55.415 | -18.897 | 16×16×16 |
| Vascular endothelial growth factor A | VEGFA | P15692 | 5t89 | -67.137 | 31.282 | 34.862 | 14×14×12 |
| Macrophage colony-stimulating factor 1 | CSF1 | P09603 | 5lxf | -1.772 | -3.724 | 4.538 | 12×14×12 |
| Granulocyte colony-stimulating factor | CSF3 | P09919 | 2d9q | -55.284 | 30.737 | 46.707 | 14×14×12 |
| Granulocyte-macrophage colony-stimulating factor | CSF2 | P04141 | 2gmf | 9.087 | 11.554 | -24.564 | 14×14×14 |
| Hepatocyte Growth Factor | HGF | P14210 | 3hn4 | 6.511 | 4.564 | 38.927 | 14×14×14 |
| Interleukin-37 | IL37 | Q9NZH6 | 5hn1 | 28.177 | 27.879 | 94.120 | 14×14×14 |
| Interleukin-38 | IL38 | Q8WWZ1 | 5bow | 8.103 | -29.317 | -15.082 | 14×14×14 |
| C-C motif chemokine 7 | CCL7 | P80098 | 4zkc | 20.086 | -2.969 | 392.225 | 14×14×14 |
| Interleukin-1 receptor antagonist protein | IL1RN | P18510 | 1irp | 9.675 | 9.289 | 1.207 | 14×14×14 |
| Interleukin-2 receptor subunit alpha | IL2RA | P01589 | 2erj | 25.179 | 51.171 | -17.961 | 14×14×14 |
| Growth-regulated alpha protein | CXCL1 | P09341 | 1mgs | -7.359 | -2.367 | -12.875 | 14×14×14 |
| C-X-C motif chemokine 5 | CXCL5 | P42830 | 2mgs | -0.527 | 3.806 | -4.435 | 14×14×14 |
| C-C motif chemokine 4 | CCL4 | P13236 | 2x6l | -25.088 | -15.876 | 2.116 | 14×14×14 |
| Signal transducer and activator of transcription 3 | STAT3 | P40763 | 6nuq | -0.425 | 28.103 | 26.120 | 14×14×14 |

Table S2 List of related targets for SARS-CoV-2

| **Target protein** | **Gene name** | **Uniprot ID** | **PDBID** | **X** | **Y** | **Z** | **Vina_Box** |
| --- | --- | --- | --- | --- | --- | --- | --- |
| S glycoprotein-Initial | S | P0DTC2 | 6vyb | 232.892 | 187.911 | 266.557 | 30×30×40 |
| S glycoprotein-Alpha | S | P0DTC2 | 7lwt | 130.169 | 178.056 | 98.661 | 34×30×46 |
| S glycoprotein-Beta | S | P0DTC2 | 7lyn | 127.634 | 1177.867 | 98.98 | 40×30×48 |
| S glycoprotein-Gamma | S | P0DTC2 | 7lww | 158.525 | 128.896 | 97.395 | 34×32×50 |
| S glycoprotein-Delta | S | P0DTC2 | 7tpa | 125.475 | 178.921 | 105.947 | 32×30×40 |
| S glycoprotein-Omicron | S | P0DTC2 | 7tei | 146.962 | 189.457 | 91.192 | 40×34×54 |
| Nucleoprotein | N | P0DTC9 | 6vyo | -15.52 | 13.866 | 32.324 | 14×14×14 |
| Papain-like proteinase | rep | P0DTD1 | 6xaa | -34.623 | 25.617 | -6.918 | 14×14×14 |
| 3C-like proteinase | rep | P0DTD1 | 6lu7 | -7.361 | 16.61 | 67.947 | 14×14×14 |
| RNA-directed RNA polymerase | rep | P0DTD1 | 6xqb | 90.346 | 87.646 | 101.248 | 14×14×14 |
| Helicase | rep | P0DTD1 | 6xez | 139.395 | 207.209 | 135.458 | 14×14×14 |
| Angiotensin-converting enzyme 2 | ACE2 | Q9BYF1 | 6m0j | -35.481 | 26.176 | -11.191 | 14×14×14 |
| Basigin | BSG | P35613 | 3b5h | 11.216 | -61.738 | -35.14 | 14×14×14 |
| Dipeptidyl peptidase 4 | DPP4 | P27487 | 4l72 | -10.154 | -42.91 | -16.971 | 14×14×14 |
| Transmembrane protease serine 2 | TMPRSS2 | O15393 | SWISS-MODEL | 13.596 | -5.775 | 27.953 | 14×14×14 |
| Furin | FURIN | P09958 | 5jxj | 51.713 | -34.17 | -2.406 | 14×14×14 |
| 78 kDa glucose-regulated protein | GRP78 | P11021 | 6dfo | 2.907 | 36.503 | 35.9 | 14×14×14 |

Table S3 Results of docking studies

| **Target** | **Mol ID^a^** | **Affinity**  **(kcal/mol)** | **Target** | **Mol ID** | **Affinity**  **(kcal/mol)** | **Target** | **Mol ID** | **Affinity**  **(kcal/mol)** | **Target** | **Mol ID** | **Affinity**  **(kcal/mol)** | **Target** | **Mol ID** | **Affinity**  **(kcal/mol)** | **Target** | **Mol ID** | **Affinity**  **(kcal/mol)** |
| --- | --- | --- | --- | --- | --- | --- | --- | --- | --- | --- | --- | --- | --- | --- | --- | --- | --- |
| IL2 | M798 | -8.5 | IL7 | M2109 | -7.2 | IL1B | M937 | -7.2 | IL12A | M588 | -7.5 | IFNG | M2441 | -9.3 | IL10 | M152 | -8.2 |
| IL2 | M688 | -8.4 | IL7 | M75 | -7.2 | IL1B | M1880 | -7.2 | IL12A | M598 | -7.5 | IFNG | M674 | -9.5 | IL10 | M909 | -8.2 |
| IL2 | M1336 | -8.3 | IL7 | M616 | -7.2 | IL1B | M2427 | -7.2 | IL12A | M798 | -7.5 | IFNG | M1386 | -10 | IL10 | M1759 | -8.2 |
| IL2 | M910 | -8 | IL7 | M689 | -7.5 | CRP | M562 | -7.2 | IL12A | M831 | -7.6 | IFNG | M910 | -10.1 | IL10 | M1881 | -8.3 |
| IL2 | M907 | -7.8 | IL7 | M1053 | -7.9 | CRP | M791 | -7.9 | IL12A | M1882 | -7.6 | IFNG | M921 | -8.7 | IL10 | M163 | -8.3 |
| IL2 | M2236 | -7.8 | CCL3 | M1336 | -7.8 | CRP | M887 | -7.9 | IL12A | M892 | -7.6 | IFNG | M1555 | -8.7 | IL10 | M823 | -8.3 |
| IL2 | M246 | -7.7 | IL1B | M2368 | -7.1 | CRP | M1336 | -7.9 | IL12A | M310 | -7.6 | IFNG | M2121 | -8.8 | IL10 | M916 | -8.3 |
| IL2 | M693 | -7.7 | IL1B | M163 | -7.1 | CRP | M1484 | -8 | IL12A | M1336 | -7.6 | IL4 | M2440 | -8.8 | IL10 | M936 | -8.3 |
| IL2 | M909 | -7.7 | IL1B | M606 | -7.2 | CRP | M684 | -8.2 | IL12A | M1485 | -7.7 | IL4 | M2451 | -8.8 | IL10 | M1882 | -8.3 |
| IL2 | M2126 | -7.7 | IL1B | M1884 | -7.2 | CRP | M694 | -8.5 | IL12A | M1883 | -7.8 | IL10 | M2493 | -8.9 | IL10 | M1883 | -8.4 |
| IL2 | M589 | -7.6 | IL1B | M152 | -7.2 | CRP | M1314 | -8.8 | IL12A | M2501 | -7.8 | IL10 | M986 | -9.1 | IL10 | M65 | -8.4 |
| IL2 | M598 | -7.6 | IL1B | M405 | -7.2 | CRP | M90 | -7.6 | TNF | M2314 | -7.8 | IL10 | M984 | -9.1 | IL10 | M840 | -8.4 |
| IL2 | M616 | -7.6 | IL1B | M729 | -7.1 | CRP | M1681 | -7.6 | TNF | M1694 | -7.8 | IL10 | M2496 | -9.2 | IL10 | M989 | -8.4 |
| IL2 | M1189 | -7.6 | IL1B | M2497 | -7.3 | IL6 | M791 | -7.6 | TNF | M986 | -7.8 | IL10 | M2492 | -9.3 | IL10 | M2109 | -8.4 |
| IL2 | M1557 | -7.6 | IL1B | M589 | -7.4 | IL6 | M1336 | -7.6 | TNF | M500 | -9.2 | IL10 | M672 | -9.5 | IL10 | M2437 | -8.4 |
| IL2 | M1884 | -7.6 | IL1B | M756 | -7.4 | IL6 | M910 | -7.7 | TNF | M672 | -9.2 | IL10 | M51 | -7.5 | IL10 | M2498 | -8.5 |
| IL2 | M152 | -7.5 | IL1B | M246 | -7.4 | IL6 | M798 | -7.9 | TNF | M910 | -9.2 | IL10 | M798 | -8.1 | IL10 | M146 | -8.5 |
| IL2 | M692 | -7.5 | IL1B | M627 | -7.4 | IL12B | M1694 | -8.3 | TNF | M2126 | -9.3 | IL10 | M1884 | -8.1 | IL10 | M606 | -8.5 |
| IL2 | M783 | -7.5 | IL1B | M2436 | -7.5 | IL12B | M258 | -8.3 | TNF | M2306 | -9.3 | IL10 | M2448 | -8.2 | IL10 | M627 | -8.6 |
| IL2 | M939 | -7.5 | IL1B | M2437 | -7.5 | IL12B | M1336 | -8.7 | TNF | M2329 | -9.3 | IL10 | M246 | -8.2 | IL10 | M635 | -8.6 |
| IL2 | M1555 | -7.5 | IL1B | M693 | -7.5 | IL12B | M798 | -7.5 | TNF | M2344 | -9.3 | IL10 | M910 | -8.2 | IL10 | M729 | -8.7 |
| IL2 | M1880 | -7.5 | IL1B | M2426 | -7.9 | IL12B | M1534 | -7.5 | IFNG | M473 | -9.3 | IL10 | M2447 | -8.2 | IL10 | M2433 | -8.7 |
| IL7 | M694 | -7.1 | IL1B | M151 | -7.1 | IL12B | M1723 | -7.5 | IFNG | M1585 | -9.3 | IL10 | M151 | -8.2 | IL10 | M2497 | -8.7 |

^a^ Mol ID is the code in self-built compounds database, refer to Table S6 for details.

Table S3 Results of docking studies

| **Target** | **Mol ID** | **Affinity**  **(kcal/mol)** | **Target** | **Mol ID** | **Affinity**  **(kcal/mol)** | **Target** | **Mol ID** | **Affinity**  **(kcal/mol)** | **Target** | **Mol ID** | **Affinity**  **(kcal/mol)** | **Target** | **Mol ID** | **Affinity**  **(kcal/mol)** | **Target** | **Mol ID** | **Affinity**  **(kcal/mol)** |
| --- | --- | --- | --- | --- | --- | --- | --- | --- | --- | --- | --- | --- | --- | --- | --- | --- | --- |
| IL10 | M2501 | -8.8 | IL13 | M2109 | -7.4 | LY86 | M840 | -7.1 | LY86 | M970 | -8.1 | MyD88 | M1466 | -8.5 | TICAM1 | M2500 | -7.3 |
| IL13 | M1880 | -8.8 | IL13 | M2490 | -7.4 | LY86 | M2451 | -7.1 | LY86 | M979 | -8.1 | MyD88 | M76 | -8.6 | TBK1 | M1694 | -7.4 |
| IL13 | M823 | -8.8 | TGFB1 | M1694 | -7.4 | LY86 | M1758 | -7.1 | LY86 | M1204 | -8.2 | MyD88 | M1681 | -8.6 | TBK1 | M1336 | -7.5 |
| IL13 | M2499 | -8.9 | TGFB1 | M1831 | -7.4 | LY86 | M2109 | -7.1 | LY86 | M1291 | -8.2 | MyD88 | M1694 | -8.7 | TBK1 | M589 | -7.6 |
| IL13 | M2501 | -9 | TGFB1 | M2336 | -7.4 | LY86 | M1932 | -7.1 | LY86 | M1957 | -8.2 | MyD88 | M1723 | -8.7 | TBK1 | M792 | -7.1 |
| IL13 | M1555 | -9.2 | TGFB1 | M589 | -7.4 | LY86 | M2444 | -7.2 | LY86 | M2171 | -8.2 | MyD88 | M1817 | -8.7 | TBK1 | M310 | -7.2 |
| IL13 | M1557 | -9.3 | TGFB1 | M1672 | -7.4 | LY86 | M1189 | -7.2 | LY86 | M2439 | -8.2 | MyD88 | M1201 | -8.9 | TBK1 | M798 | -8 |
| IL13 | M2498 | -9.3 | TGFB1 | M792 | -7.5 | LY86 | M1937 | -7.2 | LY86 | M180 | -8.2 | TRAM1 | M2305 | -8.9 | TBK1 | M500 | -8 |
| IL13 | M76 | -9.5 | TGFB1 | M798 | -7.5 | LY86 | M2440 | -7.3 | LY86 | M1305 | -8.3 | TRAM1 | M1607 | -9 | TBK1 | M910 | -8 |
| IL13 | M912 | -9.7 | TGFB1 | M910 | -7.5 | LY86 | M620 | -7.3 | LY86 | M1319 | -8.3 | TICAM1 | M801 | -9.1 | TBK1 | M1884 | -8 |
| IL13 | M61 | -7.3 | TGFB1 | M2426 | -7.5 | LY86 | M1081 | -7.4 | LY86 | M1971 | -8.3 | TICAM1 | M473 | -9.2 | IKBKE | M1884 | -8 |
| IL13 | M598 | -7.3 | TGFB1 | M2427 | -7.5 | LY86 | M1457 | -7.4 | LY86 | M2039 | -8.3 | TICAM1 | M562 | -9.3 | IKBKE | M910 | -8.1 |
| IL13 | M729 | -7.3 | TGFB1 | M2437 | -7.5 | LY86 | M66 | -8 | LY86 | M2146 | -8.3 | TICAM1 | M1831 | -9.5 | IKBKE | M1053 | -8.1 |
| IL13 | M1189 | -7.3 | TGFB1 | M2490 | -7.5 | LY86 | M2445 | -7.1 | LY86 | M52 | -8.3 | TICAM1 | M554 | -9.6 | IKBKE | M611 | -8.1 |
| IL13 | M1884 | -7.3 | TGFB1 | M2498 | -7.6 | LY86 | M989 | -7.3 | LY86 | M517 | -8.3 | TICAM1 | M1844 | -9.8 | IKBKE | M1484 | -8.1 |
| IL13 | M2462 | -7.3 | TGFB1 | M121 | -7.7 | LY86 | M1119 | -7.3 | LY86 | M1038 | -8.3 | TICAM1 | M2309 | -7.1 | IKBKE | M693 | -8.2 |
| IL13 | M2506 | -7.3 | TGFB1 | M2126 | -7.8 | LY86 | M1144 | -7.9 | LY86 | M1331 | -8.4 | TICAM1 | M2498 | -7.1 | IKBKE | M2498 | -8.2 |
| IL13 | M209 | -7.3 | TGFB1 | M2236 | -7.8 | LY86 | M1318 | -8 | LY86 | M149 | -8.4 | TICAM1 | M1884 | -7.1 | IKBKE | M612 | -8.2 |
| IL13 | M668 | -7.3 | TLR3 | M84 | -7.8 | LY86 | M1325 | -8 | LY86 | M1062 | -8.4 | TICAM1 | M2490 | -7.1 | IKBKE | M731 | -8.3 |
| IL13 | M745 | -7.3 | TLR3 | M1336 | -7.9 | LY86 | M1334 | -8 | LY86 | M1503 | -8.4 | TICAM1 | M2499 | -7.2 | IKBKE | M937 | -8.3 |
| IL13 | M798 | -7.3 | TLR3 | M1371 | -7.9 | LY86 | M2031 | -8 | LY86 | M1633 | -8.4 | TICAM1 | M61 | -7.3 | IKBKE | M1883 | -8.3 |
| IL13 | M879 | -7.3 | TLR3 | M90 | -8.1 | LY86 | M2466 | -8.1 | MyD88 | M1682 | -8.4 | TICAM1 | M1314 | -7.3 | IKBKE | M2441 | -8.3 |
| IL13 | M1053 | -7.4 | TLR3 | M792 | -8.2 | LY86 | M745 | -8.1 | MyD88 | M473 | -8.5 | TICAM1 | M2336 | -7.3 | IKBKE | M2506 | -8.4 |

Table S3 Results of docking studies

| **Target** | **Mol ID** | **Affinity**  **(kcal/mol)** | **Target** | **Mol ID** | **Affinity**  **(kcal/mol)** | **Target** | **Mol ID** | **Affinity**  **(kcal/mol)** | **Target** | **Mol ID** | **Affinity**  **(kcal/mol)** | **Target** | **Mol ID** | **Affinity**  **(kcal/mol)** | **Target** | **Mol ID** | **Affinity**  **(kcal/mol)** |
| --- | --- | --- | --- | --- | --- | --- | --- | --- | --- | --- | --- | --- | --- | --- | --- | --- | --- |
| IKBKE | M587 | -8.4 | TRAF6 | M823 | -8.4 | CCL2 | M1839 | -7.6 | CXCL10 | M658 | -8.4 | NLRP3 | M2015 | -7.4 | IFNA1 | M598 | -8.1 |
| IKBKE | M627 | -8.5 | IL6ST | M674 | -8.4 | CCL2 | M907 | -7.7 | CXCL10 | M2386 | -8.8 | NLRP3 | M2111 | -7.4 | IFNA1 | M789 | -8.2 |
| IKBKE | M726 | -8.8 | IL6ST | M769 | -8.4 | CCL2 | M1466 | -7.7 | CXCL10 | M2507 | -8.9 | NLRP3 | M2336 | -7.5 | IFNA1 | M144 | -8.5 |
| IKBKE | M772 | -9.6 | IL6ST | M2500 | -8.4 | CCL2 | M1827 | -7.8 | CXCL10 | M27 | -7.1 | NLRP3 | M1046 | -7.5 | IFNA1 | M193 | -8.5 |
| IKBKE | M2411 | -9.6 | IL6ST | M2501 | -8.4 | CCL2 | M769 | -7.8 | CXCL10 | M241 | -7.1 | NLRP3 | M1081 | -7.5 | IFNA1 | M684 | -7.3 |
| IKBKE | M2448 | -9.6 | IL6ST | M693 | -8.5 | CCL2 | M808 | -7.8 | CXCL10 | M414 | -7.1 | NLRP3 | M1094 | -7.5 | IFNA1 | M776 | -7.1 |
| IKBKE | M2501 | -9.7 | IL6ST | M798 | -8.5 | CCL2 | M860 | -7.9 | CXCL10 | M526 | -7.1 | NLRP3 | M1297 | -7.5 | IFNA1 | M793 | -7.1 |
| TRAF6 | M598 | -9.7 | IL6ST | M473 | -8.5 | CXCL10 | M909 | -7.9 | CXCL10 | M589 | -7.1 | NLRP3 | M747 | -7.5 | IFNA1 | M840 | -7.1 |
| TRAF6 | M688 | -9.7 | IL6ST | M747 | -8.5 | CXCL10 | M910 | -8 | CXCL10 | M2109 | -7.2 | NLRP3 | M972 | -7.5 | IFNA2 | M2433 | -7.1 |
| TRAF6 | M1336 | -9.8 | IL6ST | M1831 | -8.5 | CXCL10 | M1484 | -8 | CXCL10 | M2428 | -7.1 | NLRP3 | M979 | -7.5 | IFNB1 | M768 | -7.1 |
| TRAF6 | M798 | -9.9 | IL6ST | M1882 | -8.5 | CXCL10 | M909 | -8.3 | CXCL10 | M2435 | -7.4 | NLRP3 | M1055 | -7.5 | IFNB1 | M684 | -7.1 |
| TRAF6 | M950 | -10 | IL6ST | M2310 | -8.6 | CXCL10 | M910 | -8.3 | CXCL10 | M2477 | -7.5 | NLRP3 | M1056 | -7.6 | IFNB1 | M1659 | -7.1 |
| TRAF6 | M791 | -10.1 | IL6ST | M2314 | -8.6 | CXCL10 | M61 | -8.3 | IL18 | M1336 | -7.8 | NLRP3 | M1143 | -7.6 | IFNB1 | M1831 | -7.1 |
| TRAF6 | M918 | -10.5 | IL8 | M253 | -8.7 | CXCL10 | M2433 | -7.9 | NLRP3 | M1882 | -7.1 | NLRP3 | M1305 | -7.6 | IFNB1 | M150 | -7.1 |
| TRAF6 | M788 | -11.3 | IL8 | M2507 | -8.7 | CXCL10 | M2437 | -8 | NLRP3 | M1883 | -7.1 | NLRP3 | M1880 | -7.6 | IFNB1 | M2490 | -7.1 |
| TRAF6 | M789 | -8.3 | IL8 | M266 | -8.8 | CXCL10 | M637 | -8 | NLRP3 | M1881 | -7.2 | NLRP3 | M2000 | -7.7 | IFNB1 | M2493 | -7.1 |
| TRAF6 | M910 | -8.3 | IL8 | M287 | -8.8 | CXCL10 | M2426 | -8 | NLRP3 | M2109 | -7.3 | NLRP3 | M2012 | -7.7 | IFNB1 | M2501 | -7.1 |
| TRAF6 | M600 | -8.3 | CCL5 | M1694 | -9.6 | CXCL10 | M2427 | -8 | NLRP3 | M1706 | -7.4 | NLRP3 | M2055 | -7.7 | IFNB1 | M598 | -7.2 |
| TRAF6 | M917 | -8.3 | CCL5 | M1336 | -7.5 | CXCL10 | M627 | -8 | NLRP3 | M2427 | -7.6 | NLRP3 | M2443 | -7.9 | IFNB1 | M693 | -7.2 |
| TRAF6 | M75 | -8.3 | CCL5 | M589 | -7.6 | CXCL10 | M508 | -8 | NLRP3 | M66 | -7.4 | IFNA1 | M1336 | -8 | IFNB1 | M860 | -7.2 |
| TRAF6 | M606 | -8.3 | CCL5 | M2126 | -7.6 | CXCL10 | M2385 | -8.1 | NLRP3 | M1758 | -7.4 | IFNA1 | M907 | -8 | IFNB1 | M1336 | -7.2 |
| TRAF6 | M610 | -8.4 | CCL2 | M1336 | -7.6 | CXCL10 | M2436 | -8.1 | NLRP3 | M1937 | -7.4 | IFNA1 | M1607 | -8 | IFNB1 | M2492 | -7.3 |
| TRAF6 | M729 | -8.4 | CCL2 | M798 | -7.6 | CXCL10 | M598 | -8.2 | NLRP3 | M991 | -7.4 | IFNA1 | M593 | -8.1 | IFNB1 | M2495 | -7.3 |

Table S3 Results of docking studies

| **Target** | **Mol ID** | **Affinity**  **(kcal/mol)** | **Target** | **Mol ID** | **Affinity**  **(kcal/mol)** | **Target** | **Mol ID** | **Affinity**  **(kcal/mol)** | **Target** | **Mol ID** | **Affinity**  **(kcal/mol)** | **Target** | **Mol ID** | **Affinity**  **(kcal/mol)** | **Target** | **Mol ID** | **Affinity**  **(kcal/mol)** |
| --- | --- | --- | --- | --- | --- | --- | --- | --- | --- | --- | --- | --- | --- | --- | --- | --- | --- |
| IFNB1 | M562 | -7.3 | IFNAR1 | M784 | -7.7 | JAK1 | M587 | -8.1 | STAT1 | M2498 | -7.7 | IL1A | M1484 | -10.2 | IL17A | M887 | -7.6 |
| IFNB1 | M801 | -7.3 | IFNAR1 | M910 | -7.7 | JAK1 | M729 | -8.1 | STAT1 | M76 | -7.7 | IL1A | M2495 | -10.3 | IL17A | M2308 | -7.8 |
| IFNB1 | M907 | -7.4 | IFNAR1 | M1097 | -7.7 | JAK1 | M792 | -8.3 | STAT1 | M235 | -7.7 | IL1A | M51 | -10.3 | IL17A | M2314 | -8.2 |
| IFNB1 | M909 | -7.4 | IFNAR1 | M1706 | -7.7 | JAK1 | M2436 | -8.9 | STAT1 | M473 | -9.8 | IL1A | M693 | -10.4 | IL17A | M144 | -8 |
| IFNB1 | M1466 | -7.4 | IFNAR1 | M1880 | -7.8 | JAK1 | M2507 | -7.2 | STAT1 | M1883 | -9.8 | IL1A | M709 | -10 | IL17A | M798 | -8 |
| IFNB1 | M2368 | -7.5 | IFNAR1 | M2433 | -7.8 | TYK2 | M798 | -7.3 | STAT1 | M2433 | -9.8 | IL1A | M1499 | -10 | IL17A | M936 | -8 |
| IFNB1 | M2497 | -7.7 | JAK1 | M798 | -7.8 | TYK2 | M405 | -7.3 | STAT1 | M2501 | -9.9 | IL1A | M2433 | -10 | IL17A | M984 | -8 |
| IFNB1 | M2498 | -8.5 | JAK1 | M589 | -7.8 | TYK2 | M310 | -7.3 | IRF9 | M1482 | -9.9 | IL1A | M589 | -10 | IL17A | M2307 | -8 |
| IFNAR1 | M121 | -8.6 | JAK1 | M1557 | -7.8 | TYK2 | M2437 | -7.3 | IRF9 | M1883 | -9.9 | IL1A | M627 | -10 | IL17A | M2311 | -8.1 |
| IFNAR1 | M879 | -8.6 | JAK1 | M531 | -7.8 | TYK2 | M693 | -7.3 | IRF9 | M473 | -9.9 | IL1A | M637 | -10 | IL17A | M2336 | -8.1 |
| IFNAR1 | M2499 | -7.1 | JAK1 | M910 | -7.8 | STAT2 | M1336 | -7.3 | IRF9 | M674 | -9.9 | IL1A | M690 | -10 | IL17A | M2342 | -8.1 |
| IFNAR1 | M2500 | -7.1 | JAK1 | M1555 | -7.8 | STAT2 | M2427 | -7.3 | IRF9 | M1387 | -9.9 | IL1A | M756 | -10 | VEGFA | M747 | -8.1 |
| IFNAR1 | M19 | -7.1 | JAK1 | M783 | -7.9 | STAT2 | M2433 | -7.3 | TRAF3 | M310 | -9.9 | IL1A | M791 | -10.1 | VEGFA | M1208 | -8.1 |
| IFNAR1 | M180 | -7.1 | JAK1 | M1883 | -7.9 | STAT2 | M729 | -7.4 | TRAF3 | M84 | -10 | IL1A | M909 | -10.1 | VEGFA | M2506 | -8.1 |
| IFNAR1 | M668 | -7.1 | JAK1 | M405 | -7.9 | STAT2 | M589 | -7.4 | TRAF3 | M1694 | -10 | IL1A | M936 | -10.2 | CSF1 | M2498 | -8.2 |
| IFNAR1 | M2498 | -7.1 | JAK1 | M1057 | -7.9 | STAT2 | M2426 | -7.4 | TRAF3 | M2433 | -10 | IL1A | M1683 | -10.3 | CSF1 | M635 | -8.2 |
| IFNAR1 | M2501 | -7.2 | JAK1 | M1706 | -7.9 | STAT2 | M1336 | -7.4 | IL1A | M1336 | -10 | IL1A | M1880 | -10.3 | CSF1 | M2501 | -8.2 |
| IFNAR1 | M73 | -7.2 | JAK1 | M1884 | -7.9 | STAT2 | M84 | -7.6 | IL1A | M910 | -10 | IL17A | M1694 | -10.3 | CSF1 | M950 | -8.3 |
| IFNAR1 | M526 | -7.2 | JAK1 | M2426 | -8 | STAT2 | M137 | -7.6 | IL1A | M798 | -10 | IL17A | M150 | -10.3 | CSF1 | M634 | -9 |
| IFNAR1 | M689 | -7.3 | JAK1 | M2427 | -8 | STAT1 | M593 | -7.6 | IL1A | M2314 | -10.1 | IL17A | M672 | -10.6 | CSF1 | M936 | -7.7 |
| IFNAR1 | M772 | -7.3 | JAK1 | M2433 | -8 | STAT1 | M2121 | -7.6 | IL1A | M1674 | -10.1 | IL17A | M986 | -10.6 | CSF1 | M2499 | -7.7 |
| IFNAR1 | M241 | -7.4 | JAK1 | M61 | -8 | STAT1 | M19 | -7.6 | IL1A | M672 | -10.2 | IL17A | M2310 | -10.7 | CSF1 | M587 | -7.7 |
| IFNAR1 | M508 | -7.8 | JAK1 | M310 | -8.1 | STAT1 | M2476 | -7.7 | IL1A | M887 | -10.2 | IL17A | M2309 | -11.5 | CSF1 | M823 | -7.9 |

Table S3 Results of docking studies

| **Target** | **Mol ID** | **Affinity**  **(kcal/mol)** | **Target** | **Mol ID** | **Affinity**  **(kcal/mol)** | **Target** | **Mol ID** | **Affinity**  **(kcal/mol)** | **Target** | **Mol ID** | **Affinity**  **(kcal/mol)** | **Target** | **Mol ID** | **Affinity**  **(kcal/mol)** | **Target** | **Mol ID** | **Affinity**  **(kcal/mol)** |
| --- | --- | --- | --- | --- | --- | --- | --- | --- | --- | --- | --- | --- | --- | --- | --- | --- | --- |
| CSF1 | M310 | -8 | CSF2 | M1336 | -8.2 | HGF | M674 | -8 | IL38 | M690 | -7.4 | CCL7 | M674 | -7.6 | IL2RA | M674 | -7.5 |
| CSF1 | M627 | -8 | CSF2 | M1557 | -8.2 | HGF | M1884 | -8 | IL38 | M815 | -7.4 | CCL7 | M907 | -7.7 | IL2RA | M593 | -7.5 |
| CSF1 | M652 | -8.1 | CSF2 | M1809 | -8.3 | HGF | M232 | -8.1 | IL38 | M310 | -7.5 | CCL7 | M627 | -7.7 | IL2RA | M792 | -7.5 |
| CSF1 | M897 | -8.2 | CSF2 | M1883 | -8.3 | HGF | M690 | -8.2 | IL38 | M791 | -7.6 | CCL7 | M589 | -7.7 | IL2RA | M590 | -7.6 |
| CSF3 | M1336 | -8.4 | CSF2 | M27 | -8.4 | HGF | M694 | -8.2 | IL38 | M938 | -7.7 | CCL7 | M709 | -7.7 | IL2RA | M647 | -7.6 |
| CSF3 | M84 | -8.4 | CSF2 | M253 | -8.4 | HGF | M815 | -8.2 | IL38 | M650 | -7.8 | CCL7 | M722 | -7.7 | IL2RA | M405 | -7.6 |
| CSF3 | M1723 | -8.6 | CSF2 | M288 | -8.5 | HGF | M266 | -8.2 | IL38 | M693 | -7.8 | CCL7 | M921 | -7.7 | IL2RA | M637 | -7.6 |
| CSF3 | M1386 | -8.6 | CSF2 | M508 | -8.6 | HGF | M729 | -8.5 | IL38 | M709 | -7.8 | CCL7 | M1880 | -7.7 | IL2RA | M658 | -7.6 |
| CSF3 | M1585 | -8.6 | HGF | M910 | -8.6 | HGF | M756 | -7.1 | IL38 | M598 | -8.3 | CCL7 | M84 | -7.7 | IL2RA | M1557 | -7.7 |
| CSF3 | M1812 | -8.8 | HGF | M593 | -8.7 | HGF | M1557 | -7.3 | IL38 | M688 | -9 | CCL7 | M571 | -7.7 | IL2RA | M1880 | -7.7 |
| CSF3 | M1371 | -8 | HGF | M908 | -10.1 | HGF | M2247 | -7.4 | IL38 | M1859 | -7.4 | CCL7 | M611 | -7.8 | IL2RA | M549 | -7.7 |
| CSF3 | M90 | -8.1 | HGF | M1499 | -7.8 | HGF | M672 | -7.4 | IL38 | M2437 | -7.4 | CCL7 | M612 | -7.8 | IL2RA | M627 | -7.7 |
| CSF2 | M1880 | -8.1 | HGF | M1555 | -7.8 | HGF | M789 | -7.4 | IL38 | M658 | -7.4 | CCL7 | M726 | -7.9 | IL2RA | M694 | -7.8 |
| CSF2 | M674 | -8.1 | HGF | M2247 | -7.8 | HGF | M791 | -7.5 | IL38 | M674 | -7.4 | CCL7 | M740 | -7.9 | IL2RA | M1555 | -7.9 |
| CSF2 | M61 | -8.1 | HGF | M910 | -7.9 | HGF | M823 | -7.6 | IL38 | M798 | -7.5 | CCL7 | M1484 | -8 | IL2RA | M2427 | -8 |
| CSF2 | M668 | -8.1 | HGF | M798 | -7.9 | HGF | M939 | -7.8 | IL38 | M939 | -7.5 | IL1RN | M910 | -8.3 | CXCL1 | M1336 | -8 |
| CSF2 | M153 | -8.1 | HGF | M907 | -7.9 | HGF | M1053 | -7.8 | IL38 | M1053 | -7.5 | IL1RN | M1882 | -7.4 | CXCL1 | M798 | -8.1 |
| CSF2 | M241 | -8.1 | HGF | M1336 | -7.9 | HGF | M1555 | -7.9 | IL38 | M1557 | -7.5 | IL1RN | M2314 | -7.5 | CXCL1 | M887 | -8.1 |
| CSF2 | M76 | -8.1 | HGF | M598 | -7.9 | HGF | M2427 | -8 | IL38 | M2433 | -7.5 | IL1RN | M1557 | -7.5 | CXCL1 | M984 | -8.1 |
| CSF2 | M101 | -8.1 | HGF | M788 | -7.9 | HGF | M2507 | -8.2 | IL38 | M2506 | -7.6 | IL1RN | M1859 | -7.5 | CXCL1 | M986 | -8.2 |
| CSF2 | M232 | -8.2 | HGF | M908 | -7.9 | IL37 | M525 | -8.3 | CCL7 | M910 | -7.6 | IL1RN | M2310 | -7.5 | CXCL1 | M610 | -8.3 |
| CSF2 | M526 | -8.2 | HGF | M616 | -7.9 | IL38 | M1694 | -8.5 | CCL7 | M909 | -7.6 | IL1RN | M2500 | -7.5 | CXCL1 | M910 | -8.4 |
| CSF2 | M773 | -8.2 | HGF | M909 | -8 | IL38 | M1466 | -7.4 | CCL7 | M587 | -7.6 | IL2RA | M589 | -7.5 | CXCL1 | M1694 | -8.8 |

Table S3 Results of docking studies

| **Target** | **Mol ID** | **Affinity**  **(kcal/mol)** | **Target** | **Mol ID** | **Affinity**  **(kcal/mol)** | **Target** | **Mol ID** | **Affinity**  **(kcal/mol)** | **Target** | **Mol ID** | **Affinity**  **(kcal/mol)** | **Target** | **Mol ID** | **Affinity**  **(kcal/mol)** | **Target** | **Mol ID** | **Affinity**  **(kcal/mol)** |
| --- | --- | --- | --- | --- | --- | --- | --- | --- | --- | --- | --- | --- | --- | --- | --- | --- | --- |
| CXCL1 | M1826 | -7.7 | CCL4 | M1839 | -7.7 | ACE2 | M887 | -8.3 | DPP4 | M610 | -8.2 | Helicase | M549 | -7.6 | PLpro | M101 | -7.6 |
| CXCL1 | M792 | -7.8 | STAT3 | M1694 | -7.7 | ACE2 | M909 | -8.5 | DPP4 | M798 | -8.2 | Helicase | M1371 | -7.6 | PLpro | M772 | -7.6 |
| CXCL1 | M917 | -7.8 | STAT3 | M1767 | -7.7 | ACE2 | M1831 | -7.6 | DPP4 | M887 | -8.2 | Helicase | M1336 | -7.7 | PLpro | M2436 | -7.6 |
| CXCL1 | M1674 | -7.8 | STAT3 | M90 | -7.7 | ACE2 | M791 | -7.6 | DPP4 | M986 | -8.2 | Helicase | M1846 | -7.9 | PLpro | M879 | -7.6 |
| CCL4 | M986 | -7.8 | STAT3 | M473 | -7.7 | ACE2 | M1672 | -7.6 | DPP4 | M1882 | -8.2 | Helicase | M798 | -8 | PLpro | M2437 | -7.7 |
| CCL4 | M1882 | -7.8 | STAT3 | M1828 | -7.8 | ACE2 | M2492 | -7.6 | FURIN | M1694 | -8.3 | Helicase | M1692 | -7.2 | PLpro | M912 | -7.7 |
| CCL4 | M588 | -7.8 | STAT3 | M1883 | -7.8 | ACE2 | M936 | -7.6 | FURIN | M1336 | -8.3 | Helicase | M1607 | -7.2 | PLpro | M916 | -7.7 |
| CCL4 | M887 | -7.8 | 3CLpro | M540 | -7.8 | ACE2 | M2307 | -7.6 | FURIN | M1723 | -8.3 | Helicase | M473 | -7.2 | PLpro | M310 | -7.9 |
| CCL4 | M2126 | -7.8 | 3CLpro | M910 | -7.8 | ACE2 | M2329 | -7.6 | FURIN | M1883 | -8.3 | Helicase | M670 | -7.2 | PLpro | M2426 | -8 |
| CCL4 | M918 | -7.9 | 3CLpro | M473 | -7.8 | ACE2 | M635 | -7.7 | FURIN | M910 | -8.3 | Helicase | M729 | -7.2 | PLpro | M2462 | -8.4 |
| CCL4 | M1826 | -8 | 3CLpro | M918 | -7.8 | ACE2 | M688 | -7.7 | FURIN | M1682 | -8.4 | Helicase | M731 | -7.2 | PLpro | M2500 | -8.4 |
| CCL4 | M1827 | -8 | 3CLpro | M1739 | -7.8 | ACE2 | M768 | -7.7 | FURIN | M798 | -8.4 | Helicase | M823 | -7.2 | RdRp | M500 | -8.5 |
| CCL4 | M2345 | -8 | 3CLpro | M1694 | -7.8 | ACE2 | M1314 | -7.7 | FURIN | M950 | -8.5 | Helicase | M2310 | -7.3 | RdRp | M1336 | -8.5 |
| CCL4 | M1466 | -8.1 | 3CLpro | M1831 | -7.8 | ACE2 | M1827 | -8.1 | GRP78 | M910 | -8.5 | Helicase | M791 | -7.3 | RdRp | M1694 | -8.6 |
| CCL4 | M1884 | -8.1 | 3CLpro | M2121 | -7.9 | CD147 | M310 | -8.1 | GRP78 | M668 | -8.6 | Helicase | M1499 | -7.3 | TMPRSS2 | M253 | -9.1 |
| CCL4 | M2343 | -8.1 | ACE2 | M709 | -7.9 | CD147 | M562 | -8.1 | GRP78 | M909 | -9.3 | Helicase | M1826 | -7.4 | TMPRSS2 | M674 | -8.6 |
| CCL4 | M405 | -8.3 | ACE2 | M783 | -7.9 | CD147 | M672 | -8.1 | GRP78 | M598 | -7.1 | Helicase | M2312 | -7.4 | TMPRSS2 | M405 | -8.7 |
| CCL4 | M808 | -8.3 | ACE2 | M672 | -7.9 | DPP4 | M1694 | -8.1 | GRP78 | M908 | -7.1 | PLpro | M1706 | -7.4 | TMPRSS2 | M473 | -8.7 |
| CCL4 | M1846 | -8.3 | ACE2 | M801 | -7.9 | DPP4 | M1336 | -8.1 | GRP78 | M783 | -7.1 | PLpro | M909 | -7.4 | TMPRSS2 | M1884 | -8.8 |
| CCL4 | M1881 | -8.5 | ACE2 | M910 | -8 | DPP4 | M910 | -8.1 | Helicase | M1336 | -7.2 | PLpro | M2433 | -7.4 | S glycoprotein-Initial | M798 | -8.9 |
| CCL4 | M2312 | -8.5 | ACE2 | M1336 | -8.2 | DPP4 | M984 | -8.1 | Helicase | M647 | -7.2 | PLpro | M61 | -7.5 | S glycoprotein-Initial | M1336 | -8.9 |
| CCL4 | M670 | -8.6 | ACE2 | M562 | -8.2 | DPP4 | M672 | -8.1 | Helicase | M1485 | -7.2 | PLpro | M593 | -7.5 | S glycoprotein-Initial | M688 | -9 |
| CCL4 | M984 | -9.4 | ACE2 | M1683 | -8.2 | DPP4 | M909 | -8.1 | Helicase | M525 | -7.2 | PLpro | M2441 | -7.5 | S glycoprotein-Initial | M791 | -9.4 |

Table S3 Results of docking studies

| **Target** | **Mol ID** | **Affinity**  **(kcal/mol)** | **Target** | **Mol ID** | **Affinity**  **(kcal/mol)** | **Target** | **Mol ID** | **Affinity**  **(kcal/mol)** |
| --- | --- | --- | --- | --- | --- | --- | --- | --- |
| S glycoprotein-Initial | M823 | -9.2 | S glycoprotein-Gamma | M1694 | -9.9 | S glycoprotein-Delta | M887 | -7.6 |
| S glycoprotein-Initial | M917 | -9.2 | S glycoprotein-Gamma | M692 | -9.9 | S glycoprotein-Omicron | M1336 | -7.6 |
| S glycoprotein-Initial | M84 | -9.2 | S glycoprotein-Gamma | M1336 | -9.9 | S glycoprotein-Omicron | M910 | -7.7 |
| S glycoprotein-Initial | M635 | -9.2 | S glycoprotein-Gamma | M2309 | -9.9 | S glycoprotein-Omicron | M1827 | -7.7 |
| S glycoprotein-Initial | M668 | -9.2 | S glycoprotein-Gamma | M2490 | -10 | S glycoprotein-Omicron | M150 | -7.7 |
| S glycoprotein-Initial | M610 | -9.2 | S glycoprotein-Gamma | M540 | -10 | S glycoprotein-Omicron | M1607 | -7.8 |
| S glycoprotein-Initial | M936 | -9.2 | S glycoprotein-Gamma | M791 | -7.1 | S glycoprotein-Omicron | M1336 | -8 |
| S glycoprotein-Alpha | M1336 | -9.3 | S glycoprotein-Gamma | M907 | -7.1 | S glycoprotein-Omicron | M1694 | -8.2 |
| S glycoprotein-Alpha | M1839 | -9.3 | S glycoprotein-Gamma | M936 | -7.3 | S glycoprotein-Omicron | M986 | -8.3 |
| S glycoprotein-Alpha | M1694 | -9.3 | S glycoprotein-Gamma | M2491 | -8.9 | S glycoprotein-Omicron | M1883 | -8.5 |
| S glycoprotein-Alpha | M688 | -9.3 | S glycoprotein-Gamma | M688 | -8.9 | S glycoprotein-Omicron | M2433 | -9.7 |
| S glycoprotein-Alpha | M986 | -9.3 | S glycoprotein-Gamma | M792 | -8.9 | S glycoprotein-Omicron | M1839 | -9.7 |
| S glycoprotein-Beta | M1336 | -9.4 | S glycoprotein-Delta | M798 | -8.9 | S glycoprotein-Omicron | M2342 | -9.7 |
| S glycoprotein-Beta | M1683 | -9.4 | S glycoprotein-Delta | M909 | -8.9 |  |  |  |
| S glycoprotein-Beta | M1694 | -9.4 | S glycoprotein-Delta | M589 | -8.9 |  |  |  |
| S glycoprotein-Beta | M1532 | -9.5 | S glycoprotein-Delta | M1880 | -9 |  |  |  |
| S glycoprotein-Beta | M798 | -9.5 | S glycoprotein-Delta | M2433 | -9.1 |  |  |  |
| S glycoprotein-Beta | M1827 | -9.5 | S glycoprotein-Delta | M798 | -9.1 |  |  |  |
| S glycoprotein-Beta | M1839 | -9.6 | S glycoprotein-Delta | M1694 | -9.2 |  |  |  |
| S glycoprotein-Beta | M588 | -9.6 | S glycoprotein-Delta | M540 | -9.2 |  |  |  |
| S glycoprotein-Beta | M688 | -9.6 | S glycoprotein-Delta | M588 | -9.5 |  |  |  |
| S glycoprotein-Beta | M1466 | -9.8 | S glycoprotein-Delta | M791 | -10.6 |  |  |  |

Table S4 Medicinal properties of LHQW active compounds

| **Compound** | **MW** | **HBD** | **HBA** | **LogP** | **RB** | **Caco2** | **Intestinal absorption (human)** |
| --- | --- | --- | --- | --- | --- | --- | --- |
| M19 | 326.352 | 4 | 2 | 2.0199 | 1 | 0.965 | 93.931 |
| M27 | 254.241 | 4 | 2 | 2.18162 | 0 | 1.096 | 97.076 |
| M51 | 412.702 | 1 | 0 | 8.233 | 6 | 1.207 | 97.686 |
| M52 | 218.259 | 1 | 1 | 3.8693 | 0 | 1.077 | 93.234 |
| M61 | 278.267 | 4 | 3 | 2.3638 | 0 | 1.065 | 88.803 |
| M65 | 414.718 | 1 | 1 | 8.0248 | 6 | 1.18 | 93.658 |
| M66 | 194.318 | 1 | 0 | 3.8822 | 3 | 1.507 | 94.695 |
| M73 | 327.38 | 5 | 1 | 1.9382 | 2 | 1.241 | 92.476 |
| M75 | 262.268 | 3 | 2 | 3.1906 | 1 | 0.812 | 91.589 |
| M76 | 262.268 | 3 | 2 | 3.1906 | 1 | 0.775 | 91.864 |
| M84 | 593.558 | 13 | 7 | -0.6828 | 7 | -0.22 | 38.598 |
| M90 | 610.565 | 15 | 8 | -1.1566 | 7 | -0.157 | 39.128 |
| M101 | 248.241 | 3 | 0 | 2.5385 | 0 | 1.292 | 97.163 |
| M121 | 350.374 | 5 | 1 | 2.7393 | 3 | 1.018 | 92.58 |
| M137 | 684.688 | 16 | 9 | -2.4001 | 12 | -0.296 | 0 |
| M144 | 426.729 | 1 | 1 | 8.0248 | 1 | 1.208 | 95.208 |
| M146 | 414.718 | 1 | 1 | 8.0248 | 6 | 1.18 | 93.658 |
| M149 | 278.436 | 1 | 1 | 5.6605 | 13 | 1.305 | 90.273 |
| M150 | 424.713 | 1 | 0 | 8.233 | 1 | 1.219 | 97.733 |
| M151 | 412.702 | 1 | 1 | 7.8008 | 5 | 1.21 | 94.73 |
| M152 | 400.691 | 1 | 1 | 7.6347 | 5 | 1.185 | 94.209 |
| M153 | 270.24 | 5 | 3 | 1.88722 | 0 | 0.848 | 73.395 |
| M163 | 386.664 | 1 | 1 | 7.3887 | 5 | 1.186 | 94.269 |
| M180 | 297.31 | 4 | 2 | 2.9021 | 3 | 1.106 | 93.033 |
| M193 | 350.374 | 5 | 1 | 3.2206 | 3 | 1.087 | 91.498 |
| M209 | 246.306 | 3 | 2 | 2.44742 | 1 | 1.228 | 91.41 |
| M232 | 256.257 | 4 | 3 | 2.24702 | 0 | 1.191 | 96.006 |
| M235 | 306.27 | 7 | 6 | 1.2517 | 1 | -0.471 | 60.14 |
| M241 | 298.294 | 5 | 2 | 2.49864 | 1 | 1.19 | 96.517 |
| M246 | 314.469 | 2 | 0 | 4.7235 | 1 | 1.55 | 98.351 |
| M253 | 284.223 | 5 | 3 | 1.5714 | 1 | -0.137 | 62.003 |
| M258 | 862.746 | 18 | 12 | -1.0956 | 9 | -1.874 | 0 |
| M266 | 273.264 | 4 | 2 | 2.78692 | 1 | 1.119 | 95.053 |
| M287 | 270.24 | 5 | 3 | 1.3655 | 1 | -0.105 | 78.15 |
| M288 | 284.267 | 5 | 2 | 2.19022 | 1 | 1.097 | 97.655 |
| M310 | 416.382 | 9 | 5 | -0.34528 | 3 | 0.475 | 47.419 |
| M405 | 432.381 | 10 | 6 | -0.63968 | 3 | -0.571 | 40.397 |
| M414 | 268.268 | 4 | 1 | 2.48462 | 1 | 1.196 | 97.077 |
| M473 | 442.376 | 10 | 7 | 2.5276 | 3 | -1.055 | 58.969 |
| M500 | 611.529 | 15 | 10 | -1.5746 | 6 | -0.667 | 25.961 |

Table S4 Medicinal properties of LHQW active compounds

| **Compound** | **MW** | **HBD** | **HBA** | **LogP** | **RB** | **Caco2** | **Intestinal absorption (human)** |
| --- | --- | --- | --- | --- | --- | --- | --- |
| M508 | 254.241 | 4 | 2 | 2.18162 | 0 | 1.074 | 96.603 |
| M517 | 239.25 | 2 | 1 | 3.2781 | 1 | 1.31 | 92.879 |
| M525 | 287.247 | 5 | 4 | 2.3949 | 1 | 0.761 | 81.589 |
| M526 | 284.267 | 5 | 2 | 2.19022 | 1 | 1.226 | 96.289 |
| M531 | 412.702 | 1 | 1 | 7.9449 | 5 | 1.198 | 94.753 |
| M540 | 822.942 | 13 | 8 | 2.2456 | 7 | -0.812 | 0 |
| M549 | 284.267 | 5 | 1 | 2.7306 | 0 | 1.22 | 96.932 |
| M554 | 456.711 | 2 | 2 | 7.0895 | 2 | 1.285 | 100 |
| M562 | 456.711 | 2 | 2 | 7.2336 | 1 | 1.172 | 98.956 |
| M571 | 255.249 | 3 | 2 | 2.9837 | 1 | 1.131 | 92.527 |
| M587 | 408.494 | 5 | 3 | 4.7925 | 5 | 0.958 | 93.878 |
| M588 | 470.694 | 3 | 2 | 6.4126 | 1 | 0.573 | 98.222 |
| M589 | 390.479 | 4 | 1 | 5.7916 | 3 | 1.062 | 93.512 |
| M590 | 406.522 | 4 | 1 | 6.4277 | 6 | 0.977 | 89.79 |
| M593 | 354.358 | 6 | 3 | 3.3465 | 1 | 1.21 | 85.59 |
| M598 | 322.36 | 4 | 2 | 4.1781 | 3 | 1.087 | 93.337 |
| M600 | 437.512 | 5 | 3 | 5.7153 | 6 | 1.003 | 88.658 |
| M606 | 320.432 | 2 | 0 | 5.42184 | 2 | 1.532 | 96.583 |
| M610 | 353.35 | 5 | 3 | 3.8735 | 1 | 0.948 | 89.446 |
| M611 | 339.367 | 4 | 3 | 4.198 | 3 | 1.028 | 94.558 |
| M612 | 340.375 | 5 | 3 | 3.2838 | 3 | 0.965 | 95.461 |
| M616 | 324.376 | 4 | 2 | 4.0007 | 1 | 1.249 | 91.866 |
| M620 | 224.259 | 2 | 0 | 3.1423 | 4 | 1.462 | 96.961 |
| M627 | 392.495 | 4 | 2 | 5.8217 | 5 | 0.976 | 91.388 |
| M634 | 323.368 | 3 | 2 | 4.4924 | 3 | 1.022 | 91.978 |
| M635 | 390.479 | 4 | 2 | 5.8886 | 5 | 1.028 | 92.044 |
| M637 | 340.375 | 5 | 3 | 4.0186 | 3 | 1.068 | 90.991 |
| M647 | 355.366 | 5 | 4 | 3.9036 | 3 | 1.056 | 81.057 |
| M650 | 369.393 | 5 | 4 | 4.2937 | 4 | 1.035 | 84.064 |
| M652 | 407.486 | 4 | 3 | 5.7067 | 5 | 0.946 | 91.266 |
| M658 | 312.365 | 4 | 4 | 3.7833 | 2 | 1.209 | 92.609 |
| M668 | 354.358 | 6 | 3 | 3.3465 | 1 | 1.417 | 78.931 |
| M670 | 454.695 | 2 | 2 | 7.1537 | 1 | 1.179 | 95.741 |
| M672 | 454.695 | 3 | 1 | 6.6843 | 0 | 1.196 | 96.083 |
| M674 | 322.36 | 4 | 1 | 4.1861 | 0 | 1.33 | 96.287 |
| M684 | 456.711 | 3 | 2 | 6.3203 | 1 | 1.347 | 94.215 |
| M688 | 390.479 | 4 | 1 | 5.4793 | 1 | 1.526 | 94.267 |
| M689 | 299.258 | 5 | 2 | 2.7124 | 1 | 0.986 | 94.687 |
| M690 | 324.376 | 4 | 2 | 4.0007 | 1 | 1.267 | 93.317 |

Table S4 Medicinal properties of LHQW active compounds

| **Compound** | **MW** | **HBD** | **HBA** | **LogP** | **RB** | **Caco2** | **Intestinal absorption (human)** |
| --- | --- | --- | --- | --- | --- | --- | --- |
| M692 | 324.376 | 4 | 2 | 4.313 | 3 | 0.997 | 93.268 |
| M693 | 322.36 | 4 | 2 | 4.215 | 1 | 1.242 | 91.867 |
| M694 | 337.351 | 4 | 2 | 4.1679 | 1 | 0.749 | 94.176 |
| M709 | 392.495 | 4 | 2 | 5.5094 | 3 | 1.198 | 93.832 |
| M722 | 256.257 | 4 | 2 | 2.8043 | 1 | 1.207 | 91.921 |
| M726 | 324.376 | 4 | 2 | 4.313 | 3 | 0.986 | 92.214 |
| M729 | 367.377 | 5 | 1 | 4.6227 | 1 | 1.095 | 94.713 |
| M731 | 369.393 | 5 | 2 | 4.0959 | 2 | 0.926 | 95.626 |
| M740 | 354.402 | 5 | 2 | 4.2248 | 3 | 0.959 | 93.909 |
| M745 | 240.258 | 3 | 2 | 2.9939 | 3 | 1.28 | 90.62 |
| M747 | 354.402 | 5 | 2 | 4.0093 | 2 | 1.084 | 93.844 |
| M756 | 354.402 | 5 | 2 | 4.0093 | 2 | 1.116 | 94.55 |
| M768 | 456.711 | 2 | 2 | 7.2336 | 1 | 1.156 | 96.805 |
| M769 | 420.505 | 5 | 1 | 5.7034 | 3 | 1.434 | 96.025 |
| M772 | 295.314 | 3 | 0 | 3.80622 | 2 | 1.36 | 99.358 |
| M773 | 253.277 | 2 | 1 | 3.58652 | 1 | 1.279 | 94.962 |
| M776 | 424.537 | 5 | 2 | 5.4374 | 4 | 0.923 | 95.924 |
| M783 | 421.469 | 5 | 3 | 5.3822 | 3 | 0.968 | 91.975 |
| M784 | 338.403 | 4 | 1 | 4.5192 | 3 | 1.213 | 94.284 |
| M788 | 368.385 | 6 | 2 | 3.6495 | 2 | 1.083 | 93.963 |
| M789 | 371.365 | 6 | 4 | 2.7637 | 1 | 1.058 | 81.022 |
| M791 | 389.471 | 3 | 1 | 5.971 | 3 | 1.276 | 93.931 |
| M792 | 406.478 | 5 | 2 | 4.7624 | 3 | 1.166 | 94.363 |
| M793 | 337.351 | 4 | 2 | 3.8633 | 2 | 0.947 | 93.745 |
| M798 | 388.463 | 4 | 0 | 5.7615 | 1 | 1.557 | 96.935 |
| M801 | 456.711 | 2 | 2 | 7.0895 | 1 | 1.173 | 99.284 |
| M808 | 470.694 | 3 | 2 | 6.4126 | 1 | 0.573 | 98.222 |
| M815 | 338.403 | 4 | 1 | 4.3037 | 2 | 1.528 | 95 |
| M823 | 365.361 | 5 | 1 | 4.7033 | 1 | 1.092 | 94.493 |
| M831 | 368.385 | 6 | 2 | 3.6495 | 2 | 1.057 | 93.512 |
| M840 | 321.42 | 3 | 1 | 4.0431 | 6 | 1.425 | 92.341 |
| M860 | 484.721 | 4 | 1 | 6.501 | 1 | 0.635 | 96.699 |
| M879 | 281.287 | 3 | 0 | 3.4978 | 2 | 1.36 | 99.182 |
| M887 | 454.695 | 3 | 1 | 6.6843 | 0 | 1.27 | 96.817 |
| M892 | 368.385 | 6 | 2 | 3.6495 | 2 | 1.057 | 93.512 |
| M897 | 369.393 | 5 | 3 | 4.2066 | 4 | 0.932 | 88.095 |
| M907 | 406.478 | 5 | 2 | 5.2464 | 6 | 1.006 | 92.506 |
| M908 | 338.359 | 5 | 2 | 3.7377 | 4 | 0.954 | 91.334 |

Table S4 Medicinal properties of LHQW active compounds

| **Compound** | **MW** | **HBD** | **HBA** | **LogP** | **RB** | **Caco2** | **Intestinal absorption (human)** |
| --- | --- | --- | --- | --- | --- | --- | --- |
| M909 | 406.478 | 5 | 2 | 5.2464 | 6 | 0.982 | 91.28 |
| M910 | 404.462 | 5 | 1 | 5.2163 | 4 | 1.08 | 93.799 |
| M912 | 284.267 | 5 | 2 | 2.172 | 2 | 1.357 | 96.642 |
| M916 | 324.376 | 4 | 1 | 4.3469 | 3 | 1.35 | 94.981 |
| M917 | 367.377 | 5 | 2 | 4.1765 | 2 | 0.944 | 94.302 |
| M918 | 371.365 | 6 | 4 | 2.7637 | 1 | 1.096 | 80.654 |
| M921 | 442.552 | 6 | 3 | 4.6322 | 5 | 1.273 | 90.472 |
| M936 | 391.487 | 3 | 2 | 6.0011 | 5 | 1.025 | 91.79 |
| M937 | 339.367 | 4 | 3 | 4.198 | 3 | 0.959 | 91.659 |
| M938 | 338.359 | 5 | 2 | 3.6409 | 1 | 1.048 | 96.881 |
| M939 | 352.386 | 5 | 1 | 3.9439 | 2 | 1.192 | 98.495 |
| M950 | 423.485 | 5 | 4 | 5.4123 | 5 | 1.035 | 83.676 |
| M970 | 204.357 | 0 | 0 | 4.7252 | 1 | 1.408 | 93.329 |
| M972 | 204.357 | 0 | 0 | 4.5591 | 0 | 1.406 | 94.181 |
| M979 | 178.234 | 0 | 0 | 3.993 | 0 | 1.521 | 95.733 |
| M984 | 428.745 | 1 | 1 | 8.2488 | 0 | 1.225 | 94.927 |
| M986 | 426.729 | 1 | 0 | 8.457 | 0 | 1.236 | 97.452 |
| M989 | 219.287 | 1 | 1 | 4.5834 | 2 | 1.536 | 92.83 |
| M991 | 204.357 | 0 | 0 | 4.415 | 0 | 1.415 | 95.087 |
| M1038 | 204.357 | 0 | 0 | 5.2015 | 6 | 1.424 | 93.601 |
| M1046 | 215.34 | 1 | 0 | 4.02212 | 1 | 1.566 | 95.977 |
| M1053 | 337.307 | 5 | 0 | 4.056 | 2 | 1.044 | 100 |
| M1055 | 215.34 | 1 | 0 | 3.64002 | 0 | 1.519 | 95.434 |
| M1056 | 204.357 | 0 | 0 | 4.415 | 0 | 1.415 | 94.831 |
| M1057 | 370.357 | 7 | 1 | 2.3341 | 2 | 1.328 | 99.393 |
| M1062 | 204.357 | 0 | 0 | 4.7252 | 1 | 1.402 | 93.109 |
| M1081 | 204.357 | 0 | 0 | 4.2709 | 1 | 1.396 | 95.385 |
| M1094 | 202.297 | 1 | 0 | 3.2681 | 0 | 1.501 | 97.786 |
| M1097 | 264.321 | 4 | 1 | 1.9636 | 1 | 1.16 | 96.106 |
| M1119 | 202.341 | 0 | 0 | 4.84492 | 4 | 1.561 | 94.168 |
| M1143 | 220.356 | 1 | 0 | 3.6739 | 0 | 1.213 | 95.183 |
| M1144 | 236.355 | 2 | 0 | 3.5532 | 4 | 1.348 | 97.456 |
| M1189 | 207.252 | 0 | 0 | 4.3809 | 1 | 1.573 | 95.46 |
| M1201 | 428.434 | 10 | 6 | -2.0204 | 7 | -0.385 | 26.44 |
| M1204 | 284.311 | 4 | 2 | 2.8969 | 5 | 1.046 | 94.992 |
| M1208 | 354.311 | 8 | 6 | -0.6459 | 4 | -0.607 | 18.192 |
| M1291 | 212.248 | 2 | 0 | 3.0436 | 3 | 1.42 | 96.762 |
| M1297 | 204.357 | 0 | 0 | 4.5811 | 1 | 1.434 | 96.105 |
| M1305 | 204.357 | 0 | 0 | 4.2709 | 1 | 1.396 | 95.385 |
| M1314 | 472.71 | 3 | 3 | 6.206 | 2 | 0.475 | 97.713 |

Table S4 Medicinal properties of LHQW active compounds

| **Compound** | **MW** | **HBD** | **HBA** | **LogP** | **RB** | **Caco2** | **Intestinal absorption (human)** |
| --- | --- | --- | --- | --- | --- | --- | --- |
| M1318 | 204.357 | 0 | 0 | 4.2709 | 1 | 1.407 | 96.505 |
| M1319 | 204.357 | 0 | 0 | 4.7252 | 0 | 1.421 | 94.732 |
| M1325 | 204.357 | 0 | 0 | 4.2709 | 1 | 1.412 | 97.485 |
| M1331 | 204.357 | 0 | 0 | 4.5811 | 1 | 1.419 | 96.178 |
| M1334 | 250.47 | 0 | 0 | 6.4876 | 7 | 1.401 | 92.021 |
| M1336 | 540.48 | 8 | 5 | 5.7787 | 4 | 0.481 | 88.2 |
| M1371 | 595.53 | 14 | 9 | -1.2802 | 6 | -0.635 | 30.32 |
| M1386 | 516.455 | 11 | 7 | 1.0296 | 7 | -0.674 | 25.972 |
| M1387 | 516.455 | 11 | 7 | 1.0296 | 7 | -0.674 | 25.972 |
| M1457 | 204.357 | 0 | 0 | 4.8913 | 4 | 1.414 | 93.87 |
| M1466 | 432.645 | 4 | 2 | 4.7646 | 0 | 1.175 | 94.493 |
| M1482 | 516.455 | 11 | 7 | 1.0296 | 7 | -0.606 | 23.574 |
| M1484 | 516.455 | 12 | 7 | 0.4639 | 6 | -0.953 | 45.701 |
| M1485 | 516.455 | 12 | 7 | 0.4639 | 6 | -1.202 | 36.468 |
| M1499 | 449.388 | 10 | 7 | -0.132 | 4 | -0.448 | 45.79 |
| M1503 | 255.249 | 3 | 2 | 2.9837 | 1 | 1.075 | 93.397 |
| M1532 | 1043.251 | 20 | 12 | -0.2986 | 10 | 427.461 | -2.816 |
| M1534 | 1075.249 | 22 | 13 | -1.3962 | 11 | -1.037 | 0 |
| M1555 | 272.388 | 2 | 2 | 3.6092 | 0 | 1.184 | 92.539 |
| M1557 | 270.372 | 2 | 1 | 3.8174 | 0 | 1.199 | 95.064 |
| M1585 | 457.432 | 12 | 7 | -3.10802 | 7 | -0.339 | 10.107 |
| M1607 | 472.71 | 4 | 2 | 5.855 | 1 | 1.261 | 93.731 |
| M1633 | 182.222 | 1 | 0 | 3.1661 | 2 | 1.674 | 96.161 |
| M1659 | 430.717 | 2 | 1 | 8.84026 | 12 | 1.212 | 89.679 |
| M1672 | 468.766 | 2 | 0 | 8.7397 | 1 | 1.187 | 93.506 |
| M1674 | 454.695 | 2 | 1 | 7.2977 | 2 | 1.391 | 97.429 |
| M1681 | 640.591 | 16 | 10 | -1.525 | 10 | -0.458 | 8.723 |
| M1682 | 624.592 | 15 | 9 | -1.0159 | 10 | -0.613 | 15.621 |
| M1683 | 468.766 | 2 | 0 | 8.7397 | 1 | 1.209 | 95.939 |
| M1692 | 486.781 | 3 | 1 | 8.1006 | 5 | 1.176 | 94.309 |
| M1694 | 504.45 | 8 | 6 | 5.08104 | 0 | -0.451 | 100 |
| M1706 | 369.373 | 7 | 1 | 2.4354 | 1 | 1.047 | 97.066 |
| M1723 | 622.576 | 15 | 8 | -0.4794 | 7 | -0.342 | 36.306 |
| M1739 | 449.388 | 10 | 7 | 0.6012 | 3 | -0.481 | 55.911 |
| M1758 | 426.685 | 2 | 1 | 6.6696 | 4 | 1.313 | 96.2 |
| M1759 | 166.264 | 1 | 0 | 2.7677 | 0 | 1.417 | 96.762 |
| M1767 | 316.485 | 2 | 2 | 6.2835 | 12 | 1.625 | 87.239 |
| M1809 | 478.45 | 11 | 7 | 0.1323 | 8 | -0.338 | 37.831 |
| M1812 | 638.575 | 16 | 9 | -1.507 | 8 | -1.385 | 17.738 |

Table S4 Medicinal properties of LHQW active compounds

| **Compound** | **MW** | **HBD** | **HBA** | **LogP** | **RB** | **Caco2** | **Intestinal absorption (human)** |
| --- | --- | --- | --- | --- | --- | --- | --- |
| M1817 | 610.565 | 15 | 9 | -1.4044 | 10 | -0.605 | 14.796 |
| M1826 | 426.729 | 1 | 1 | 8.0248 | 0 | 1.226 | 95.227 |
| M1827 | 468.766 | 2 | 0 | 8.5956 | 1 | 1.22 | 97.569 |
| M1828 | 666.849 | 11 | 8 | 1.4061 | 4 | -0.634 | 31.899 |
| M1831 | 472.71 | 3 | 3 | 6.0603 | 1 | 0.496 | 98.076 |
| M1839 | 496.732 | 4 | 0 | 7.2551 | 1 | 1.177 | 97.511 |
| M1844 | 472.71 | 3 | 3 | 6.0603 | 2 | 0.507 | 99.179 |
| M1846 | 458.727 | 3 | 1 | 6.9492 | 2 | 1.171 | 94.882 |
| M1859 | 318.457 | 2 | 1 | 4.7753 | 4 | 1.411 | 96.419 |
| M1880 | 311.337 | 5 | 0 | 2.6802 | 0 | 1.91 | 94.232 |
| M1881 | 383.4 | 7 | 0 | 3.0895 | 2 | 1.062 | 92.885 |
| M1882 | 369.373 | 7 | 1 | 2.4354 | 1 | 1.147 | 92.435 |
| M1883 | 367.357 | 7 | 0 | 2.5847 | 1 | 1.111 | 97.985 |
| M1884 | 287.322 | 2 | 1 | 3.0962 | 0 | 1.309 | 93.67 |
| M1932 | 170.255 | 0 | 0 | 3.5612 | 0 | 1.412 | 96.7 |
| M1937 | 192.261 | 0 | 0 | 4.30142 | 0 | 1.524 | 95.962 |
| M1957 | 269.344 | 3 | 1 | 3.1926 | 5 | 1.415 | 94.122 |
| M1971 | 204.357 | 0 | 0 | 4.7252 | 3 | 1.386 | 94.507 |
| M2000 | 200.325 | 0 | 0 | 4.54162 | 1 | 1.565 | 95.569 |
| M2012 | 198.309 | 0 | 0 | 4.53164 | 1 | 1.433 | 95.648 |
| M2015 | 202.341 | 0 | 0 | 4.63192 | 1 | 1.55 | 94.935 |
| M2031 | 204.357 | 0 | 0 | 5.0354 | 4 | 1.42 | 94 |
| M2039 | 204.357 | 0 | 0 | 5.2015 | 6 | 1.424 | 93.601 |
| M2055 | 204.357 | 0 | 0 | 4.7252 | 1 | 1.406 | 93.474 |
| M2109 | 192.261 | 0 | 0 | 4.30142 | 0 | 1.454 | 96.448 |
| M2111 | 231.271 | 2 | 1 | 3.1198 | 2 | 1.286 | 94.428 |
| M2121 | 433.389 | 9 | 6 | 0.8956 | 3 | 0.257 | 57.843 |
| M2126 | 424.669 | 2 | 1 | 7.022 | 0 | 1.184 | 93.94 |
| M2146 | 269.344 | 3 | 1 | 3.1926 | 5 | 1.415 | 94.122 |
| M2171 | 242.274 | 3 | 2 | 2.2146 | 1 | 1.239 | 94.555 |
| M2236 | 400.691 | 1 | 1 | 8.4055 | 5 | 1.183 | 92.06 |
| M2247 | 244.246 | 4 | 0 | 2.66352 | 1 | 1.318 | 96.171 |
| M2305 | 410.73 | 0 | 0 | 9.054 | 1 | 1.246 | 97.283 |
| M2306 | 412.702 | 1 | 0 | 8.0669 | 1 | 1.232 | 98.086 |
| M2307 | 428.745 | 1 | 1 | 8.2488 | 1 | 1.229 | 97.631 |
| M2308 | 410.73 | 0 | 0 | 8.9099 | 1 | 1.257 | 98.122 |
| M2309 | 428.745 | 1 | 1 | 8.2488 | 1 | 1.229 | 97.631 |
| M2310 | 384.648 | 1 | 0 | 7.4308 | 0 | 1.229 | 96.918 |
| M2311 | 412.702 | 1 | 0 | 8.0669 | 1 | 1.232 | 98.086 |

Table S4 Medicinal properties of LHQW active compounds

| **Compound** | **MW** | **HBD** | **HBA** | **LogP** | **RB** | **Caco2** | **Intestinal absorption (human)** |
| --- | --- | --- | --- | --- | --- | --- | --- |
| M2312 | 424.713 | 1 | 0 | 8.233 | 1 | 1.495 | 97.768 |
| M2314 | 382.676 | 0 | 0 | 8.4179 | 0 | 1.257 | 95.696 |
| M2329 | 410.73 | 0 | 0 | 9.054 | 1 | 1.252 | 98.201 |
| M2336 | 410.73 | 0 | 0 | 9.1981 | 1 | 1.284 | 99.251 |
| M2342 | 410.73 | 0 | 0 | 9.054 | 1 | 1.251 | 96.328 |
| M2343 | 410.73 | 0 | 0 | 9.054 | 1 | 1.268 | 97.486 |
| M2344 | 412.702 | 1 | 0 | 8.0669 | 1 | 1.243 | 100 |
| M2345 | 410.73 | 0 | 0 | 9.054 | 1 | 1.267 | 97.486 |
| M2368 | 398.675 | 1 | 1 | 7.4107 | 4 | 1.209 | 94.138 |
| M2385 | 307.305 | 4 | 1 | 3.145 | 2 | 1.137 | 97.642 |
| M2386 | 265.268 | 3 | 2 | 3.2729 | 1 | 1.236 | 95.483 |
| M2411 | 315.301 | 5 | 2 | 3.0009 | 3 | 1.195 | 94.097 |
| M2426 | 208.213 | 4 | 1 | 1.587 | 3 | 1.201 | 93.567 |
| M2427 | 293.278 | 4 | 1 | 3.296 | 1 | 1.265 | 96.769 |
| M2428 | 263.252 | 3 | 1 | 3.2874 | 0 | 1.261 | 96.549 |
| M2433 | 323.348 | 5 | 1 | 3.4127 | 2 | 1.234 | 94.456 |
| M2435 | 277.279 | 4 | 1 | 3.1604 | 1 | 1.258 | 97.58 |
| M2436 | 291.306 | 4 | 0 | 3.4634 | 2 | 1.308 | 100 |
| M2437 | 305.289 | 5 | 0 | 3.1835 | 1 | 1.299 | 100 |
| M2439 | 321.332 | 5 | 0 | 3.472 | 3 | 1.291 | 100 |
| M2440 | 273.288 | 4 | 4 | 1.7759 | 4 | 0.805 | 92.053 |
| M2441 | 257.289 | 3 | 3 | 1.8556 | 4 | 0.993 | 91.446 |
| M2443 | 325.364 | 4 | 1 | 2.6489 | 3 | 1.285 | 96.716 |
| M2444 | 311.337 | 4 | 2 | 2.3459 | 2 | 1.038 | 95.381 |
| M2445 | 225.291 | 1 | 1 | 2.6591 | 4 | 1.83 | 93.349 |
| M2447 | 506.602 | 4 | 2 | 4.6122 | 11 | 0.946 | 91.31 |
| M2448 | 444.531 | 4 | 2 | 3.3183 | 10 | 1.088 | 92.407 |
| M2451 | 239.274 | 2 | 2 | 2.7929 | 3 | 1.234 | 91.667 |
| M2462 | 198.306 | 2 | 0 | 3.2852 | 10 | 1.595 | 95.163 |
| M2466 | 255.317 | 2 | 2 | 2.02 | 5 | 1.27 | 91.887 |
| M2476 | 344.387 | 5 | 3 | 2.7711 | 2 | 1.179 | 94.757 |
| M2477 | 352.346 | 6 | 3 | 3.04437 | 3 | .0.539 | 92.1 |
| M2490 | 354.358 | 6 | 0 | 3.2192 | 2 | 1.181 | 96.852 |
| M2491 | 354.358 | 6 | 0 | 3.2192 | 2 | 1.655 | 99.844 |
| M2492 | 428.701 | 2 | 1 | 7.2038 | 6 | 1.503 | 95.598 |
| M2493 | 412.702 | 1 | 0 | 8.233 | 6 | 1.353 | 98.066 |
| M2495 | 426.685 | 2 | 0 | 7.412 | 6 | 1.172 | 97.098 |
| M2496 | 428.701 | 2 | 0 | 7.4919 | 6 | 1.179 | 96.38 |
| M2497 | 428.701 | 2 | 1 | 7.2038 | 6 | 1.417 | 96.049 |
| M2498 | 344.363 | 6 | 0 | 2.3471 | 2 | 1.233 | 98.297 |

Table S4 Medicinal properties of LHQW active compounds

| **Compound** | **MW** | **HBD** | **HBA** | **LogP** | **RB** | **Caco2** | **Intestinal absorption (human)** |
| --- | --- | --- | --- | --- | --- | --- | --- |
| M2499 | 346.379 | 6 | 1 | 2.1389 | 2 | 1.15 | 95.867 |
| M2500 | 346.379 | 6 | 1 | 2.1405 | 3 | 1.128 | 95.806 |
| M2501 | 330.38 | 5 | 0 | 3.1681 | 2 | 1.355 | 96.73 |
| M2506 | 282.299 | 4 | 2 | 1.7448 | 2 | 1.02 | 95.29 |
| M2507 | 293.278 | 4 | 2 | 2.842 | 1 | 0.983 | 94.508 |

Table S5 Results of docking studies-Affinity range of the compounds corresponding to each target

| **Target** | **Final compound quantity** | **Affinity range**  **(kcal/mol)** | **Target** | **Final compound quantity** | **Affinity range**  **(kcal/mol)** | **Target** | **Final compound quantity** | **Affinity range**  **(kcal/mol)** |
| --- | --- | --- | --- | --- | --- | --- | --- | --- |
| IL2 | 22 | -7.5~-8.5 | CCL5 | 4 | -7.1~-7.8 | CCL7 | 18 | 7.7~-8.1 |
| IL7 | 6 | -7.1~-7.9 | CCL2 | 12 | -7~-7.6 | IL1RN | 7 | -8.2~-9.3 |
| CCL3 | 1 | -7.8 | CXCL10 | 25 | -7.5~-8.5 | IL2RA | 16 | -7.1~-8 |
| IL1B | 20 | -7~-7.9 | IL18 | 1 | -7.3 | CXCL1 | 0 | -7 |
| CRP | 10 | -7~-7.2 | NLRP3 | 29 | -7.1~-8.6 | CXCL5 | 20 | -7.3~-8 |
| IL6 | 4 | -8~-8.8 | IFNA1 | 12 | -7.1~-7.8 | CCL4 | 6 | -8.4~-9.1 |
| IL12B | 6 | -7.7~8.7 | IFNA2 | 1 | -7 | STAT3 | 8 | -8.7~-9.4 |
| IL12A | 11 | -7.6~-7.8 | IFNB1 | 22 | -7.8~-8.9 | ACE2 | 22 | -9.3~-10 |
| TNF | 10 | -9.3~-10.1 | IFNAR1 | 21 | -7.3~-7.7 | CD147 | 3 | -7.1~-7.3 |
| IFNG | 9 | -8.8~-9.5 | JAK1 | 22 | -9.9~-10.4 | DPP4 | 11 | -8.9~-10.6 |
| IL4 | 2 | --7~7.5 | TYK2 | 11 | -10.1~-11.5 | FURIN | 8 | -7.7~-8.5 |
| IL10 | 38 | -8.2~-9.7 | STAT1 | 3 | -7.6~-8.2 | GRP78 | 12 | -9.7~-10.6 |
| IL13 | 24 | -7.4~-8.2 | STAT2 | 11 | -8.1~-9 | Helicase | 15 | -9.4~-9.8 |
| TGFB1 | 16 | -7~-8 | IRF9 | 5 | -7.9~-8.2 | N Protein | 0 | -7 |
| TLR3 | 5 | -7~-7.3 | TRAF3 | 4 | -8.6~-8.8 | PLpro | 17 | -7.6~-8.2 |
| LY86 | 44 | -8~-9.8 | IL1A | 24 | -8.1~-10.1 | RdRp | 3 | -7.9~-8.1 |
| MyD88 | 9 | -7.2~-7.6 | IL17A | 17 | -7.9~-8.5 | TMPRSS2 | 5 | -8.2~-8.8 |
| TRAM1 | 2 | -7.1~-7.2 | VEGFA | 3 | -7~-7.1 | S glycoprotein-Initial | 11 | -9.4~-10.4 |
| TICAM1 | 15 | -8.1~-8.8 | CSF1 | 13 | -7.3~-8.5 | S glycoprotein-Alpha | 5 | -8.4~-9.3 |
| TBK1 | 9 | -9.7~-11.3 | CSF3 | 8 | -7.6~-9 | S glycoprotein- Beta | 10 | -8.4~-9.4 |
| IKBKE | 20 | -8.4~-9.6 | CSF2 | 29 | -7.5~-8.3 | S glycoprotein- Gamma | 17 | -8.5~-8.7 |
| TRAF6 | 17 | -7.6~-8.3 | HGF | 30 | -7.5~-8.8 | S glycoprotein- Delta | 11 | -7.9~-8.7 |
| IL6ST | 12 | -8~-8.9 | IL37 | 1 | -7 | S glycoprotein- Omicron | 7 | -8.4~-8.7 |
| CXCL8 | 4 | -7~-7.2 | IL1F10 | 22 | -7.8~-8.5 |  |  |  |

Table S6 Compounds database of LHQW

| **Structure Type** | **Mol ID** | **Molecule Name** | **Source** | **Reference** | **Structure Type** | **Mol ID** | **Molecule Name** | **Source** | **Reference** |
| --- | --- | --- | --- | --- | --- | --- | --- | --- | --- |
| Flavonoid | M84 | Linarin | BLG，YXC | TCMSP; [[1](#_ENREF_1)] | Flavonoid | M598 | (E)-1-(2,4-dihydroxyphenyl)-3-(2,2-dimethylchromen-6-yl)prop-2-en-1-one | GC | TCMSP |
| Flavonoid | M90 | Neohesperidin | BLG | TCMSP; [[1](#_ENREF_1)] | Flavonoid | M600 | Kanzonol K | GC | TCMSP |
| Flavonoid | M235 | Gallocatechin | DH，MH | TCMSP; [[2](#_ENREF_2)] | Flavonoid | M606 | Licoagropin | GC | TCMSP |
| Flavonoid | M345 | Kaempferol | DH，GC，HJT，JYH，LQ，MH，MMGZ，YXC,KXR | Shanghai Institute of Organic Chemistry of CAS, TCMSP ; [[3-6](#_ENREF_3)] | Flavonoid | M610 | Semilicoisoflavone B | GC | TCMSP[[7](#_ENREF_7)] |
| Flavonoid | M473 | (−)-Epicatechin-3-O-gallate | DH | [[3](#_ENREF_3)] | Flavonoid | M611 | Glepidotin A | GC | TCMSP |
| Flavonoid | M500 | Rutin | DH，GC，HJT，JYH，LQ，MH，YXC | TCMSP ,TCMID [[2](#_ENREF_2), [3](#_ENREF_3), [6](#_ENREF_6), [8](#_ENREF_8), [9](#_ENREF_9)] | Flavonoid | M612 | Glepidotin B | GC | TCMSP |
| Flavonoid | M517 | Flavonol | DH | [[4](#_ENREF_4)] | Flavonoid | M616 | Phaseollinisoflavan | GC | TCMSP |
| Flavonoid | M518 | Quercetin | DH，GC，GHX，HJT0，JYH，LQ，MH，YXC | TCMSP,TCMID[[4](#_ENREF_4), [6](#_ENREF_6), [10](#_ENREF_10)] | Flavonoid | M620 | Dibenzoylmethane | GC | TCMSP |
| Flavonoid | M525 | Luteolin | DH，JYH，LQ，MH，YXC | TCMSP[[6](#_ENREF_6), [9](#_ENREF_9)] | Flavonoid | M627 | Glabrol | GC | TCMSP |
| Flavonoid | M549 | Maackiain | GC | TCMSP | Flavonoid | M634 | Licoflavone A | GC | TCMSP[[11](#_ENREF_11)] |
| Flavonoid | M558 | Glycyrol | GC，KXR | TCMSP[[7](#_ENREF_7), [11](#_ENREF_11)] | Flavonoid | M635 | 7-Hydroxy-2-[4-hydroxy-3-(3-methylbut-2-enyl)phenyl]-6-(3-methylbut-2-enyl)chromone | GC | TCMSP |
| Flavonoid | M571 | 7,4'-Dihydroxyflavone | GC | TCMSP | Flavonoid | M637 | Licoflavanone | GC | TCMSP[[11](#_ENREF_11)] |
| Flavonoid | M587 | 3-Hydroxyglabrol | GC | TCMSP | Flavonoid | M647 | Gancaonin L | GC | TCMSP |
| Flavonoid | M589 | (2S)-2-[4-hydroxy-3-(3-methylbut-2-enyl)phenyl]-8,8-dimethyl-2,3-dihydropyrano[2,3-f]chromen-4-one | GC | TCMSP | Flavonoid | M650 | Gancaonin O | GC | TCMSP |
| Flavonoid | M590 | Euchrenone | GC | TCMSP | Flavonoid | M652 | Gancaonin Q | GC | TCMSP |
| Flavonoid | M593 | Glyasperin F | GC | TCMSP | Flavonoid | M658 | Gancaonin V | GC | TCMSP |

Table S6 Compounds database of LHQW

| **Structure Type** | **Mol ID** | **Molecule Name** | **Source** | **Reference** | **Structure Type** | **Mol ID** | **Molecule Name** | **Source** | **Reference** |
| --- | --- | --- | --- | --- | --- | --- | --- | --- | --- |
| Flavonoid | M668 | Licoisoflavanone | GC | TCMSP[[11](#_ENREF_11)] | Flavonoid | M756 | 3'-Methoxyglabridin | GC | TCMSP |
| Flavonoid | M674 | Shinpterocarpin | GC | TCMSP | Flavonoid | M769 | Kanzonol F | GC | TCMSP |
| Flavonoid | M688 | Hispaglabridin B | GC | TCMSP[[7](#_ENREF_7), [11](#_ENREF_11)] | Flavonoid | M772 | 7-Acetoxy-2-methylisoflavone | GC | TCMSP |
| Flavonoid | M689 | Glyzaglabrin | GC | TCMSP[[11](#_ENREF_11)] | Flavonoid | M773 | 7-Hydroxy-2-methyl-3-phenyl-chromone | GC | TCMSP |
| Flavonoid | M690 | Glabridin | GC，KXR | TCMSP[[7](#_ENREF_7), [11](#_ENREF_11)] | Flavonoid | M776 | Kanzonol H | GC | TCMSP |
| Flavonoid | M692 | Glabranin | GC | TCMSP[[11](#_ENREF_11)] | Flavonoid | M783 | Gancaonin H | GC | TCMSP[[11](#_ENREF_11)] |
| Flavonoid | M693 | Glabrene | GC | TCMSP[[7](#_ENREF_7), [11](#_ENREF_11)] | Flavonoid | M784 | Licoagrocarpin | GC | TCMSP |
| Flavonoid | M694 | Glabrone | GC | TCMSP[[7](#_ENREF_7), [11](#_ENREF_11)] | Flavonoid | M788 | Glyasperin M | GC | TCMSP |
| Flavonoid | M709 | Hispaglabridin A | GC | TCMSP[[7](#_ENREF_7), [11](#_ENREF_11)] | Flavonoid | M789 | Glycyrrhiza flavonol A | GC | TCMSP |
| Flavonoid | M722 | (2R)-7-hydroxy-2-(4-hydroxyphenyl)chroman-4-one | GC | TCMSP | Flavonoid | M791 | Kanzonol E | GC | TCMSP |
| Flavonoid | M726 | Isobavachin | GC | TCMSP | Flavonoid | M792 | Kanzonol Z | GC | TCMSP |
| Flavonoid | M729 | Isoglycyrol | GC | TCMSP[[11](#_ENREF_11)] | Flavonoid | M793 | Licoagroisoflavone | GC | TCMSP |
| Flavonoid | M731 | Isoglycycoumarin | GC | TCMSP | Flavonoid | M798 | Xambioona | GC | TCMSP[[6](#_ENREF_6)] |
| Flavonoid | M740 | 1-Methoxyphaseollidin | GC | TCMSP[[12](#_ENREF_12)] | Flavonoid | M815 | 4'-O-methylglabridin | GC | TCMID[[7](#_ENREF_7), [11](#_ENREF_11)] |
| Flavonoid | M745 | (Z)-1-(2,4-dihydroxyphenyl)-3-phenylprop-2-en-1-one | GC | TCMSP | Flavonoid | M823 | Gancaonin F | GC | TCMID[[11](#_ENREF_11)] |
| Flavonoid | M747 | 3'-Hydroxy-4'-O-Methylglabridin | GC | TCMSP | Flavonoid | M831 | Glycyrrhisoflavanone | GC | TCMID |

Table S6 Compounds database of LHQW

| **Structure Type** | **Mol ID** | **Molecule Name** | **Source** | **Reference** | **Structure Type** | **Mol ID** | **Molecule Name** | **Source** | **Reference** |
| --- | --- | --- | --- | --- | --- | --- | --- | --- | --- |
| Flavonoid | M879 | 4-Oxo-3-phenyl-4H-chromen-7-yl acetate | GC | TCMID | Flavonoid | M938 | Glabroisoflavanone A | GC | [[11](#_ENREF_11)] |
| Flavonoid | M889 | 7-Methoxy-4'-hydroxyflavone | GC | Shanghai Institute of Organic Chemistry of CAS | Flavonoid | M939 | Glabroisoflavanone B | GC | [[11](#_ENREF_11)] |
| Flavonoid | M892 | Glycyrrhisoflvanone | GC | Shanghai Institute of Organic Chemistry of CAS | Flavonoid | M950 | Isoangustone A | GC | [[7](#_ENREF_7)] |
| Flavonoid | M897 | Topazolin | GC | Shanghai Institute of Organic Chemistry of CAS | Flavonoid | M1053 | Pachyrhizin | GHX | TCMID |
| Flavonoid | M907 | Glycyrdione B | GC | Shanghai Institute of Organic Chemistry of CAS | Flavonoid | M1189 | Flavylium | HJT | TCMID |
| Flavonoid | M908 | Glyinflanin B | GC | Shanghai Institute of Organic Chemistry of CAS | Flavonoid | M1222 | Herbacetin | HJT，MH | [[2](#_ENREF_2), [13](#_ENREF_13)] |
| Flavonoid | M909 | Glyinflanin C | GC | Shanghai Institute of Organic Chemistry of CAS | Flavonoid | M1336 | Ochnaflavone | JYH | TCMSP[[10](#_ENREF_10)] |
| Flavonoid | M910 | Glyinflanin D | GC | Shanghai Institute of Organic Chemistry of CAS | Flavonoid | M1371 | Lonicerin | JYH1 | TCMSP[[10](#_ENREF_10)] |
| Flavonoid | M912 | Gancaonin K | GC | Shanghai Institute of Organic Chemistry of CAS | Flavonoid | M1499 | Luteolin-7-O-β-d-galactoside | JYH | [[10](#_ENREF_10)] |
| Flavonoid | M916 | Asperopterocaepin | GC | [[12](#_ENREF_12)] | Flavonoid | M1503 | Chrysin | JYH | [[10](#_ENREF_10)] |
| Flavonoid | M917 | Glycyrrhiza isoflavone B | GC | [[12](#_ENREF_12)] | Flavonoid | M1654 | Myricetin | KXR，LQ，YXC | TCMSP [[5](#_ENREF_5), [8](#_ENREF_8)] |
| Flavonoid | M918 | Glycyrrhizaisoflavone A | GC | [[12](#_ENREF_12)] | Flavonoid | M1751 | Euxanthone | LQ | TCMSP |
| Flavonoid | M919 | Glycyrrhizaisoflavone C | GC | [[12](#_ENREF_12)] | Flavonoid | M2121 | Afzelin | MH，YXC | TCMID[[2](#_ENREF_2), [6](#_ENREF_6), [8](#_ENREF_8)] |
| Flavonoid | M921 | Glycyuralin A | GC | [[12](#_ENREF_12)] | Flavonoid | M2171 | Leucoanthpcyanin | MH | [[2](#_ENREF_2)] |
| Flavonoid | M936 | Licoflavone B | GC | [[11](#_ENREF_11)] | Flavonoid | M2411 | Pilloin | YXC | [[6](#_ENREF_6)] |
| Flavonoid | M937 | Licoflavone C | GC | [[11](#_ENREF_11)] | Terpenoid | M66 | Dihydro-β-ionone | BLG，LQ，MH | TCMSP |

Table S6 Compounds database of LHQW

| **Structure Type** | **Mol ID** | **Molecule Name** | **Source** | **Reference** | **Structure Type** | **Mol ID** | **Molecule Name** | **Source** | **Reference** |
| --- | --- | --- | --- | --- | --- | --- | --- | --- | --- |
| Terpenoid | M144 | Lupeol | BLG | TCMSP | Terpenoid | M972 | β-Patchoulene | GHX | TCMSP[[14](#_ENREF_14), [15](#_ENREF_15)] |
| Terpenoid | M150 | Lupenone | BLG | TCMSP | Terpenoid | M984 | Epi-Friedelanol | GHX | TCMSP |
| Terpenoid | M209 | Manicol | BLG | [[1](#_ENREF_1)] | Terpenoid | M986 | Friedelin | GHX | TCMSP |
| Terpenoid | M540 | Glycyrrhizin | DH，GC | TCMSP[[7](#_ENREF_7), [9](#_ENREF_9), [11](#_ENREF_11), [12](#_ENREF_12)] | Terpenoid | M991 | α-Patchoulene | GHX | TCMSP[[14](#_ENREF_14), [15](#_ENREF_15)] |
| Terpenoid | M554 | Betulinic acid | GC，KXR，LQ | TCMSP[[16](#_ENREF_16)] | Terpenoid | M1038 | α-Farnesene | GHX，JYH，MH | TCMID,TCMSP |
| Terpenoid | M562 | Oleanolic acid | GC，GHX，JYH，LQ，MH，YXC | TCMSP[[6](#_ENREF_6), [10](#_ENREF_10)] | Terpenoid | M1056 | Patrinene | GHX | TCMID |
| Terpenoid | M588 | 18β-Glycyrrhetinic acid | GC | TCMSP[[7](#_ENREF_7), [12](#_ENREF_12)] | Terpenoid | M1062 | Valencene | GHX | TCMID |
| Terpenoid | M670 | Licorice-saponin C2_qt | GC | TCMSP | Terpenoid | M1081 | α-Copaene | GHX | [[15](#_ENREF_15)] |
| Terpenoid | M905 | Glyyunnansapogenin G | GC | Shanghai Institute of Organic Chemistry of CAS | Terpenoid | M1532 | Loniceroside A | JYH | [[10](#_ENREF_10)] |
| Terpenoid | M672 | Licorice-saponin F3_qt | GC | TCMSP | Terpenoid | M1094 | 14-Nor-β-patchoul-1(5),2-diene-4-one | GHX | [[15](#_ENREF_15)] |
| Terpenoid | M684 | Glycyrrhetol | GC | TCMSP | Terpenoid | M1097 | Pocahemiketal A | GHX | [[15](#_ENREF_15)] |
| Terpenoid | M768 | 11-Deoxyglycyrrhetic acid | GC | TCMSP | Terpenoid | M1119 | α-Curcumene | GHX，MMGZ | [[15](#_ENREF_15), [17](#_ENREF_17)] |
| Terpenoid | M801 | Ursolic acid | GC，JYH，KXR，LQ022，MH24，YXC223 | TCMSP[[16](#_ENREF_16)] | Terpenoid | M1143 | α-Patchoulone | GHX | [[15](#_ENREF_15)] |
| Terpenoid | M808 | 18α-Glycyrrhetinic acid | GC | TCMID | Terpenoid | M1144 | 7-Epichabrolidione A | GHX | [[15](#_ENREF_15)] |
| Terpenoid | M860 | Methylglycyrrhetate | GC | TCMID | Terpenoid | M1297 | γ-Muurolene | JYH0 | TCMSP |
| Terpenoid | M887 | Deoxyglabrolide | GC | Shanghai Institute of Organic Chemistry of CAS | Terpenoid | M1305 | Copaene | JYH | TCMSP |
| Terpenoid | M970 | δ-Guaiene | GHX，MH | TCMSP[[14](#_ENREF_14), [15](#_ENREF_15)] | Terpenoid | M1314 | Hederagenol | JYH0 | TCMSP[[10](#_ENREF_10)] |

Table S6 Compounds database of LHQW

| **Structure Type** | **Mol ID** | **Molecule Name** | **Source** | **Reference** | **Structure Type** | **Mol ID** | **Molecule Name** | **Source** | **Reference** |
| --- | --- | --- | --- | --- | --- | --- | --- | --- | --- |
| Terpenoid | M1318 | α-Cubebene | JYH | TCMSP | Terpenoid | M1844 | Alphitolic acid | LQ | [[16](#_ENREF_16)] |
| Terpenoid | M1319 | Bicyclogermacrene | JYH | TCMSP | Terpenoid | M1846 | Ocotillone | LQ | [[16](#_ENREF_16)] |
| Terpenoid | M1325 | β-Cubebene | JYH | TCMSP | Terpenoid | M1850 | Dammar-24-ene-3β-acetate-20S-ol | LQ | [[16](#_ENREF_16)] |
| Terpenoid | M1331 | (1R,4S,4aR)-1-isopropyl-4-methyl-7-methylene-2,3,4,4a,5,6-hexahydro-1H-naphthalene | JYH | TCMSP | Terpenoid | M1852 | Garcinielliptone Q | LQ | [[16](#_ENREF_16)] |
| Terpenoid | M1457 | Zingiberene | JYH | TCMSP | Terpenoid | M1853 | Agatholic acid | LQ | [[16](#_ENREF_16)] |
| Terpenoid | M1466 | Chlorogenin | JYH，YXC | TCMID,TCMSP | Terpenoid | M1859 | 3-Oxoanticopalic acid | LQ | [[16](#_ENREF_16)] |
| Terpenoid | M1534 | Loniceroside C | JYH | [[10](#_ENREF_10)] | Terpenoid | M1932 | 3,7-Dimethyl-cyclopenta cyclooctene | MH | TCMSP |
| Terpenoid | M1607 | Ziziphin_qt | KXR1 | TCMSP | Terpenoid | M1971 | Trans-α-Bergamotene | MH | TCMSP |
| Terpenoid | M1659 | α-Tocopherol | KXR | [[5](#_ENREF_5)] | Terpenoid | M2000 | α-Calacorene | MH | TCMSP |
| Terpenoid | M1683 | β-Amyrin acetate | LQ | TCMSP[[16](#_ENREF_16)] | Terpenoid | M2012 | Guaiazulene | MH | TCMSP |
| Terpenoid | M1695 | D-Limonene | LQ，YXC | TCMSP[[16](#_ENREF_16)] | Terpenoid | M2015 | (+)-Calamenene | MH | TCMSP |
| Terpenoid | M1826 | ψ-Taraxasterol | LQ | [[16](#_ENREF_16)] | Terpenoid | M2031 | β-Bisabolene | MH | TCMSP |
| Terpenoid | M1827 | Taraxasterol acetate | LQ | [[16](#_ENREF_16)] | Terpenoid | M2039 | (Z,E)-α-Farnesene | MH | TCMSP |
| Terpenoid | M1828 | Nigaichigoside F1 | LQ | [[16](#_ENREF_16)] | Terpenoid | M2055 | Acoradiene | MH | TCMSP |
| Terpenoid | M1831 | Corosolic acid | LQ | [[16](#_ENREF_16)] | Terpenoid | M2126 | Maragenin ii | MH | TCMID |
| Terpenoid | M1839 | 3β-Acetoxy-11-en-olean-28,13-olide | LQ | [[16](#_ENREF_16)] | Terpenoid | M2305 | Diploptene-b | MMGZ | [[17](#_ENREF_17)] |

Table S6 Compounds database of LHQW

| **Structure Type** | **Mol ID** | **Molecule Name** | **Source** | **Reference** | **Structure Type** | **Mol ID** | **Molecule Name** | **Source** | **Reference** |
| --- | --- | --- | --- | --- | --- | --- | --- | --- | --- |
| Terpenoid | M2307 | Diplopterol | MMGZ | [[17](#_ENREF_17)] | Alkaloid | M75 | Indigo | BLG | TCMSP[[1](#_ENREF_1)] |
| Terpenoid | M2308 | Filicen | MMGZ | [[17](#_ENREF_17)] | Alkaloid | M76 | Indirubin | BLG | TCMSP[[1](#_ENREF_1)] |
| Terpenoid | M2309 | Hydroxyhopane | MMGZ | [[17](#_ENREF_17), [18](#_ENREF_18)] | Alkaloid | M101 | Tryptanthrin | BLG | TCMSP[[1](#_ENREF_1)] |
| Terpenoid | M2310 | 17-α-H-trisnorhopanone | MMGZ | [[17](#_ENREF_17)] | Alkaloid | M1880 | Suspensine A | LQ | [[16](#_ENREF_16)] |
| Terpenoid | M2312 | Fern-9(11)-en-12-one | MMGZ | [[17](#_ENREF_17)] | Alkaloid | M1881 | (−)-7ʹ-O-methylegenine | LQ | [[16](#_ENREF_16)] |
| Terpenoid | M2314 | Fern-9(11)-ene | MMGZ | [[17](#_ENREF_17)] | Alkaloid | M1882 | (−)-Rgenine | LQ | [[16](#_ENREF_16)] |
| Terpenoid | M2329 | Diploptene | MMGZ | TCMSP | Alkaloid | M1883 | (−)-Bicuculline | LQ | [[16](#_ENREF_16)] |
| Terpenoid | M2342 | Filicene | MMGZ | TCMSP | Alkaloid | M1884 | Rutaecarpine | LQ | [[16](#_ENREF_16)] |
| Terpenoid | M2343 | 7-Fernene | MMGZ | TCMSP | Alkaloid | M2378 | Cepharadione B | YXC | TCMID[[6](#_ENREF_6)] |
| Terpenoid | M2345 | Fernene | MMGZ | Shanghai Institute of Organic Chemistry of CAS | Alkaloid | M2385 | Norcepharadione b | YXC | TCMID |
| Terpenoid | M2497 | 3-Hydroxy-β-sitoster-5-en-7-one | YXC | [[6](#_ENREF_6)] | Alkaloid | M2386 | Piperolactam A | YXC | TCMID[[6](#_ENREF_6)] |
| Terpenoid | M2498 | Strigone | YXC | [[6](#_ENREF_6)] | Alkaloid | M2426 | Aristolactam I | YXC | [[6](#_ENREF_6)] |
| Terpenoid | M2499 | Strigol | YXC | [[6](#_ENREF_6)] | Alkaloid | M2427 | Aristolactam II | YXC | [[6](#_ENREF_6)] |
| Terpenoid | M2500 | Sorgomol | YXC | [[6](#_ENREF_6)] | Alkaloid | M2428 | Aristolactam AII | YXC | [[6](#_ENREF_6)] |
| Terpenoid | M2501 | 5-Deoxystrigol | YXC | [[6](#_ENREF_6)] | Alkaloid | M2433 | Cepharadione A | YXC | [[6](#_ENREF_6)] |
| Alkaloid | M61 | Hydroxyindirubin | BLG | TCMSP[[1](#_ENREF_1)] | Alkaloid | M2434 | Ouregidione | YXC | [[6](#_ENREF_6)] |

Table S6 Compounds database of LHQW

| **Structure Type** | **Mol ID** | **Molecule Name** | **Source** | **Reference** | **Structure Type** | **Mol ID** | **Molecule Name** | **Source** | **Reference** |
| --- | --- | --- | --- | --- | --- | --- | --- | --- | --- |
| Alkaloid | M2435 | Lysicamine | YXC | [[6](#_ENREF_6)] | Steroid | M146 | β-Sitosterol | BLG，DH，GC，HJT，JYH，KXR，LQ，MH，MMGZ，YXC | TCMSP[[2](#_ENREF_2), [5](#_ENREF_5), [12](#_ENREF_12), [17](#_ENREF_17)] |
| Alkaloid | M2436 | Atherospermidine | YXC | [[6](#_ENREF_6)] | Steroid | M151 | Stigmasterol | BLG，DH，JYH，KXR，MH，YXC | TCMSP[[5](#_ENREF_5), [6](#_ENREF_6), [9](#_ENREF_9)] |
| Alkaloid | M2437 | Liriodenine | YXC | [[6](#_ENREF_6)] | Steroid | M152 | Campesterol | BLG，MH | TCMSP |
| Alkaloid | M2439 | Houttuynamide A | YXC | [[6](#_ENREF_6)] | Steroid | M163 | Cholesterol | BLG，KXR | TCMSP[[19](#_ENREF_19)] |
| Alkaloid | M2440 | Houttuynamide B | YXC | [[6](#_ENREF_6), [20](#_ENREF_20)] | Steroid | M246 | Progesterone | DH | TCMSP |
| Alkaloid | M2441 | Houttuynamide C | YXC | [[6](#_ENREF_6), [20](#_ENREF_20)] | Steroid | M531 | Isofucosterol | DH，KXR | TCMID[[9](#_ENREF_9)] |
| Alkaloid | M2443 | 1，2-Dimethoxy-3-hydroxy-5-oxonoraporphine | YXC | [[6](#_ENREF_6)] | Steroid | M1555 | 17-β-Estradiol | KXR | TCMSP |
| Alkaloid | M2444 | N-phenethylbenzamide | YXC | [[6](#_ENREF_6)] | Steroid | M1557 | Estrone | KXR | TCMSP |
| Alkaloid | M2451 | cis-N-(4-Hydroxystyryl)benzamide | YXC | [[6](#_ENREF_6)] | Steroid | M1758 | Lactucasterol | LQ | TCMSP |
| Alkaloid | M2459 | N-Methylasimilobine | YXC | [[6](#_ENREF_6), [20](#_ENREF_20)] | Steroid | M2236 | Ergost-5-en-3β-ol | MH | [[2](#_ENREF_2)] |
| Alkaloid | M2476 | (+)-Isoboldine-beta-N-oxide | YXC | [[6](#_ENREF_6)] | Steroid | M2306 | Adianton | MMGZ | [[17](#_ENREF_17)] |
| Alkaloid | M2477 | Telitoxinone | YXC | [[6](#_ENREF_6)] | Steroid | M2311 | Isoadianton | MMGZ | [[17](#_ENREF_17)] |
| Alkaloid | M2506 | Houttuycorine | YXC | [[20](#_ENREF_20)] | Steroid | M2336 | Hopene II | MMGZ | TCMSP |
| Alkaloid | M2507 | Noraristolodione | YXC | [[20](#_ENREF_20)] | Steroid | M2344 | Adiantone | MMGZ | TCMSP |
| Steroid | M51 | 24-Ethylcholest-4-en-3-one | BLG，LQ，MH | TCMSP | Steroid | M2368 | Brassicasterol | YXC | TCMSP |
| Steroid | M65 | Clionasterol | BLG，GC，JYH，MH | TCMSP，TCMID | Steroid | M2492 | Stigmast-4-ene-3β,6β-diol | YXC | [[6](#_ENREF_6)] |

Table S6 Compounds database of LHQW

| **Structure Type** | **Mol ID** | **Molecule Name** | **Source** | **Reference** | **Structure Type** | **Mol ID** | **Molecule Name** | **Source** | **Reference** |
| --- | --- | --- | --- | --- | --- | --- | --- | --- | --- |
| Steroid | M2493 | β-Sitoster-4-en-3-one | YXC | [[6](#_ENREF_6)] | Organoheterocyclic compound | M1763 | Onjixanthone I | LQ | TCMSP |
| Steroid | M2495 | Stigmast-4-ene-3,6-dione | YXC | [[6](#_ENREF_6)] | Organoheterocyclic compound | M1334 | 1,6-Dicyclohexylhexane | JYH | TCMSP |
| Steroid | M2496 | Stigmastane-3,6-dione | YXC | [[6](#_ENREF_6)] | Organoheterocyclic compound | M1633 | 4-Phenylbenzaldehyde | KXR | [[21](#_ENREF_21)] |
| Lignan | M137 | Clemastanin B | BLG | TCMSP[[1](#_ENREF_1)] | Organoheterocyclic compound | M1759 | Matatabiether | LQ | TCMSP |
| Lignan | M1057 | Paulownin | GHX | TCMID | Organoheterocyclic compound | M2111 | Demethylsuberosin | MH | TCMID |
| Lignan | M1731 | Forsythinol | LQ | TCMSP | Organooxygen compound | M193 | Isaindigotone | BLG | [[1](#_ENREF_1)] |
| Lignan | M1748 | Olivil | LQ | TCMSP[[16](#_ENREF_16)] | Organooxygen compound | M376 | Lindleyin | DH | Shanghai Institute of Organic Chemistry of CAS[[3](#_ENREF_3)] |
| Lignan | M2490 | Epi-enschicine methyl ether | YXC | [[6](#_ENREF_6)] | Organooxygen compound | M840 | Isolobelanine | GC | TCMID |
| Lignan | M2491 | Sesamin | YXC | [[6](#_ENREF_6)] | Organooxygen compound | M1200 | Rosarin | HJT | [[13](#_ENREF_13), [22](#_ENREF_22), [23](#_ENREF_23)] |
| Lignan | M1781 | 7ʹ-Epi-8-hydroxypinoresinol | LQ | [[16](#_ENREF_16)] | Organooxygen compound | M1201 | Rosavin | HJT | [[13](#_ENREF_13), [22-24](#_ENREF_22)] |
| Organoheterocyclic compound | M19 | Isaindigodione | BLG | TCMSP[[1](#_ENREF_1)] | Organooxygen compound | M1386 | 3,4-Dicaffeoylquinic acid | JYH | TCMSP[[10](#_ENREF_10)] |
| Organoheterocyclic compound | M52 | Quindoline | BLG | TCMSP | Organooxygen compound | M1387 | 4,5-Dicaffeoylquinic acid | JYH | TCMSP[[10](#_ENREF_10)] |
| Organoheterocyclic compound | M121 | 3-[(3,5-dimethoxy-4-oxo-1-cyclohexa-2,5-dienylidene)methyl]-2,4-dihydro-1H-pyrrolo[2,1-b]quinazolin-9-one | BLG | TCMSP | Organooxygen compound | M1482 | 3,5-O-dicaffeoylquinic acid | JYH | [[10](#_ENREF_10)] |
| Organoheterocyclic compound | M266 | Toralactone | DH | TCMSP | Organooxygen compound | M1484 | 1,5-O-dicaffeoylquinic acid | JYH | [[10](#_ENREF_10)] |
| Organoheterocyclic compound | M836 | Gmelofuran | GC | TCMID | Organooxygen compound | M1485 | 1,4-O-dicaffeoylquinic acid | JYH | [[10](#_ENREF_10)] |
| Organoheterocyclic compound | M1046 | Epiguaipyridine | GHX | TCMID | Organooxygen compound | M1585 | Amygdalin | KXR | TCMSP[[5](#_ENREF_5)] |
| Organoheterocyclic compound | M1055 | Patchoulipyridine | GHX | TCMID[[15](#_ENREF_15)] | Organooxygen compound | M1673 | Isorengyol | LQ | TCMSP |

Table S6 Compounds database of LHQW

| **Structure Type** | **Mol ID** | **Molecule Name** | **Source** | **Reference** | **Structure Type** | **Mol ID** | **Molecule Name** | **Source** | **Reference** |
| --- | --- | --- | --- | --- | --- | --- | --- | --- | --- |
| Organooxygen compound | M1675 | Rengyol | LQ | TCMSP[[16](#_ENREF_16)] | Anthraquinone | M253 | Rhein | DH，MH | TCMSP[[2-4](#_ENREF_2), [9](#_ENREF_9)] |
| Organooxygen compound | M1752 | Myrcenol | LQ | TCMSP | Anthraquinone | M258 | Sennoside A | DH | TCMSP[[3](#_ENREF_3), [9](#_ENREF_9)] |
| Organooxygen compound | M1812 | Lianqiaoxinoside B | LQ | [[16](#_ENREF_16)] | Anthraquinone | M287 | Aloe-emodin | DH | TCMSP[[3](#_ENREF_3), [4](#_ENREF_4), [9](#_ENREF_9)] |
| Organooxygen compound | M2131 | Norrubrofusarin | MH | TCMID | Anthraquinone | M288 | Physcion | DH，MH | TCMSP[[2-4](#_ENREF_2), [9](#_ENREF_9)] |
| Organooxygen compound | M2462 | Perlolyrine | YXC | [[6](#_ENREF_6), [20](#_ENREF_20)] | Anthraquinone | M310 | Chrysophanol 8-o-β-d-glucopyranoside | DH | TCMID[[3](#_ENREF_3), [4](#_ENREF_4), [9](#_ENREF_9)] |
| Phenylpropanoid | M1204 | Caffeic acid phenethyl ester | HJT，MH | TCMID[[25](#_ENREF_25)] | Anthraquinone | M405 | Emodin-1-O-β-D-glucoside | DH | Shanghai Institute of Organic Chemistry of CAS[[4](#_ENREF_4)] |
| Phenylpropanoid | M1681 | Suspensaside | LQ | TCMSP[[16](#_ENREF_16)] | Anthraquinone | M414 | Chrysophanol 8-Me ether | DH | [[3](#_ENREF_3)] |
| Phenylpropanoid | M1682 | Forsythoside A | LQ，YXC | TCMSP[[6](#_ENREF_6), [16](#_ENREF_16)] | Anthraquinone | M508 | 1,8-Dihydroxy-3-methyl anthraquinone | DH | [[9](#_ENREF_9)] |
| Phenylpropanoid | M1726 | β-Hydroxyacteoside | LQ | TCMSP[[16](#_ENREF_16)] | Anthraquinone | M526 | 1,8-Dihydroxy-3-methyl-6-methoxy anthraquinone | DH | [[9](#_ENREF_9)] |
| Phenylpropanoid | M1809 | Calceolarioside B | LQ，YXC | [[6](#_ENREF_6), [16](#_ENREF_16)] | Benzenoid | M73 | Sinoacutine | BLG | TCMSP |
| Phenylpropanoid | M956 | Acteoside | GHX，LQ，YXC | TCMSP[[6](#_ENREF_6), [16](#_ENREF_16)] | Benzenoid | M180 | (E)-3-(3', 5'-dimethoxy-4'-hydroxy-benzylidene)-2-indolinone | BLG | [[1](#_ENREF_1)] |
| Phenylpropanoid | M1021 | Campneoside | GHX | TCMSP | Benzenoid | M979 | Phenanthrene | GHX，MH | TCMSP |
| Phenylpropanoid | M1817 | Calceolarioside C | LQ | [[16](#_ENREF_16)] | Benzenoid | M989 | N-Phenyl-1-naphthylamine | GHX | TCMSP |
| Anthraquinone | M27 | Crysophanol | BLG，DH | TCMSP，TCMID[[3](#_ENREF_3), [4](#_ENREF_4), [9](#_ENREF_9)] | Benzenoid | M1020 | Phenanthrone | GHX | TCMSP |
| Anthraquinone | M153 | Emodin | BLG，DH | TCMSP[[3](#_ENREF_3), [4](#_ENREF_4), [9](#_ENREF_9)] | Benzenoid | M1291 | Benzyl benzoate | HJT，JYH | TCMSP[[26](#_ENREF_26)] |
| Anthraquinone | M232 | Emodinanthrone | DH，LQ | TCMSP | Benzenoid | M1688 | Hydroxytyrosol | LQ | TCMSP[[16](#_ENREF_16)] |
| Anthraquinone | M241 | 1,8-Dihydroxy-3-methoxy-2,6-dimethyl-9,10-anthraquinone | DH | TCMSP | Benzenoid | M1767 | Urushiol III | LQ | TCMSP |

Table S6 Compounds database of LHQW

| **Structure Type** | **Mol ID** | **Molecule Name** | **Source** | **Reference** |
| --- | --- | --- | --- | --- |
| Benzenoid | M1937 | 3-Methylphenanthrene | MH | TCMSP |
| Benzenoid | M1957 | O-benzoyl-L-(+)-pseudoephedrine | MH | TCMSP |
| Benzenoid | M2109 | 3-Methyl phenanthrene | MH | TCMID |
| Benzenoid | M2146 | O-benzoyl-L-ephedrine | MH | [[2](#_ENREF_2)] |
| Benzenoid | M2247 | 1-Methyl-2, 3-methylenedioxy-6-naphthalenecarboxylic acidmethyl ester | MH | [[2](#_ENREF_2)] |
| Benzenoid | M2445 | 3,4-Dihydroxy-N-(2-phenylethyl)benzamide | YXC | [[6](#_ENREF_6)] |
| Benzenoid | M2466 | N-(1-hydroxymethyl-2-phenylethyl)benzamide | YXC | [[6](#_ENREF_6)] |
| Acid | M149 | Linolenic acid | BLG，KXR，LQ，MH，YXC | TCMSP[[1](#_ENREF_1), [2](#_ENREF_2), [6](#_ENREF_6), [27](#_ENREF_27)] |
| Acid | M2447 | Aurantiamide acetate | YXC | [[6](#_ENREF_6)] |
| Acid | M2448 | Aurantiamide | YXC | [[6](#_ENREF_6)] |

Note: LQ, Forsythiae Fructus; JYH, Lonicerae Japonicae Flos; MH, Ephedrae Herba; KXR, Armeniacae Semen Amarum; BLG, Isatidis Radix; MMGZ, Dryopteridis Crassirhizomatis Rhizoma; YXC, Houttuyniae Herba; GHX, Pogostemonis Herba; DH, Rhei Radix et Rhizoma; HJT, Rhodiolae Crenulatae Radix et Rhizoma; GC, Glycyrrhizae Radix et Rhizoma.

Table S7 Information about 14 articles related to LHQW Network Pharmacology

| Paper | Shichao Zheng, et al. , 09/04/2020  Network Pharmacology analysis of the Therapeutic Mechanisms of the traditional Chinese herbal formula Lian Hua Qing Wen in Corona virus Disease 2019 (COVID-19), gives fundamental support to the clinical use of LHQW | Qidong Xia, et al. , 09/11/2020  Network pharmacology and molecular docking analyses on Lianhua Qingwen capsule indicate Akt1 is a potential target to treat and prevent COVID-19 | Wenpan Peng, et al. , 18/12/2020  To explore the material basis and mechanism of Lianhua Qingwen Prescription against COVID-19 based on network pharmacology | Xiaobo Zhang, et al. , 24/2/2021  A network pharmacology based approach for predicting active ingredients and potential mechanism of Lianhuaqingwen capsule in treating COVID-19 | Xiaoying Ling, et al. , 02/18/2020  Exploring material basis and mechanism of Lianhua Qingwen Prescription against coronavirus based on network pharmacology | Xujie Wang, et al. , 07/10/2020  Mechanism of Lianhua Qingwen Capsule for the Treatment of Coronavirus disease 2019 (COVID-19) Based on Network Pharmacology and Chemical Composition Research | Haiyan Yan, et al. , 11/23/2020  Mechanism and material basis of Lianhua Qingwen capsule for improving clinical cure rate of COVID-19: a study based on network pharmacology and molecular docking technology | Shuang Chen, et al. , 01/27/2021  Molecular Mechanism Analysis of Lianhua Qingwen Monarch Medicine Curing COVID-19 Based on Network Pharmacology |
| --- | --- | --- | --- | --- | --- | --- | --- | --- |
| Techniques |  |  |  |  |  |  |  |  |
| Compounds source | ETCM, TCMSP | TCMSP | TCMSP | TCMSP, BATMAN, and TCMID | TCMSP | TCMSP | TCMSP | TCMSP |
| The key compounds | oleanolic acid, pseudoephedrine, luteolin, kaempferol, sitosterol, quercetin | beta-carotene, kaempferol, luteolin, naringenin, quercetin, wogonin | Quercetin, kaempferol | quercetin, luteolin, wogonin, kaempferol | Kaempferol, quercetin, luteolin, glycyrrhetinic acid, stigmasterol, indigo | Forsythiaside, isoliquiritin, emodin, rhein, kaempferol, quercetin, etc. | MOL000522, MOL004989, MOL003330, MOL001495, MOL001494, MOL004908, MOL004863, MOL001749, MOL004806, MOL001495 | Quercetin, luteolin, kaempferol, wogonin, β-sitosterol, 5-hydroxy-7-methoxy-2-(3,4,5-trimethoxyphenyl)chromone |
| Targets source | ETCM | DisGeNET, DrugBank, Genecards, OMIM, PharmGkb, Pubchem, Therapeutic Target Database (TTD) | GeneCards, Therapeutic Target Database (TTD), and Comparative Toxicogenomics Database (CTD) | GeneCards | Batman, Swiss Target Prediction, Genecards | GeneCards, DisGeNET, UniProt, PubMed | CooLGeN, GeneCards, Therapeutic Target Database (TTD) | Genecards |
| The key targets | HMGB1, PCNA, TNF, AKT1, VCAM1, PIK3CG, JAK1, ACE2 | Akt1 | 31 key targets, including IL6, TNF, etc. | IL-6、TNF、MAPK1 | PTGS2, IL6, CASP3, MAPK1, EGFR, ACE2 and so on | ACE2, IL6, IL10, TNF, CCL2, TMPRSS2, ADAM17, DPP4 and so on | MAPK1, NLRP3, HSP90AA1, TLR9, AKT1 | CASP3, CASP8, CCL2, CXCL10, CXCL8, FOS, IL10, IL1A, IL1B, IL4, IL6, MAPK1, MAPK14, MAPK8, RELA, TNF |
| Language | English | English | English | English | Chinese | Chinese | Chinese | Chinese |

Table S7 Information about 14 articles related to LHQW Network Pharmacology

| Paper | Facai Wang, et al. , 03/18/2020  Clinical Efficacy and Mechanism of Lianhua Qingwen Granule on COVID-19 Based on Network Pharmacology Research | Lin Wang, et al. , 02/29/2020  Study on the Network Pharmacology and Preliminary Evidence of Lianhua Qingwen in the treatment of novel coronavirus (2019-nCoV) Pneumonia | Xiaohui Bai, et al. , 06/04/2020  Searching for Antiviral Substances of Lianhua Qingwen Based on Network Pharmacology | Ying Ma, et al. , 18/09/2020  To explore the mechanism of lianhua qingwen capsule in the prevention and treatment of Covid-19 based on network pharmacology | Haijuan Wang, et al. , 31/08/2021  Study on the mechanism of Lianhua Qingwen capsule absorbed components in prevention and treatment of COVID-19 by intervening on cytokines storm based on network pharmacology and molecular docking | Yongfeng Zhou, et al. , 01/06/2021  Screening for Anti-Inflammation Quality Markers of Lianhua Qingwen Capsule Based on Network Pharmacology, UPLC, and Biological Activity |
| --- | --- | --- | --- | --- | --- | --- |
| Techniques |  |  |  |  |  |  |
| Compounds source | TCMSP | TCMSP, TCMID and related literatures | TCMSP, ETCM, chemtcm and related literatures | TCMSP | TCMSP | TCMSP, TDT |
| The key compounds | quercetin, luteolin, kaempferol, sitosterol | quercetin, luteolin, kaempferol, sitosterol, naringenin, locustin, aloe emodin, wogonin, daikon | ---- | Gallic acid, Rutin, Neochlorogenic acid, etc. | Emodin, Formononetin, Rutin, Galli acid and Liquiritigenin | chlorogenic acid, isochlorogenic acid B and isochlorogenic acid C |
| Targets source | Genecards, UniProt, OMIM | NCBI,GenCards | Swiss Target Prediction, | SwissTarget Prediction, GeneCards | GeneCards, OMIM, Drugbank | OMIM |
| The key targets | IL-10, CD40LG, IFNG, CCＲ5, TNF, ACE2, IL-6, CXCL8, IFNA1, CCL2, IFNB1, TMPＲSS2, CCL5, CXCL10, TP53, CD209, IL-2, CLEC4M, ACE, STAT1 | IL6, TNF, MAPK1, IL1B, MAPK8 and so on | IL6R etc. | TNF, TP53, IL6, CASP3、 IL2, MAPK14, RELA | AKT1, IL6, TP53, JUN etc. | COX-2 |
| Language | Chinese | Chinese | Chinese | Chinese | Chinese | English |

**References**

1. Zhou W, Zhang XY. Research progress of Chinese herbal medicine Radix isatidis (banlangen). Am J Chin Med. 2013;41(4):743-64.

2. Miao SM, Zhang Q, Bi XB, Cui JL, Wang ML. A review of the phytochemistry and pharmacological activities of Ephedra herb. Chin J Nat Med. 2020;18(5):321-44.

3. Zheng QX, Wu HF, Guo J, Nan HJ, Chen SL, Yang JS*, et al.* Review of Rhubarbs: Chemistry and Pharmacology. Chin Herb Med. 2013;5(1):9-32.

4. Rokaya MB, Munzbergova Z, Timsina B, Bhattarai KR. Rheum australe D. Don: a review of its botany, ethnobotany, phytochemistry and pharmacology. J Ethnopharmacol. 2012;141(3):761-74.

5. Behzad M, Saeid HS, Majid AS, Qian Y. A Systematic Review of Phytochemical and Phytotherapeutic Characteristics of Bitter Almond. Int J Pharm Phytopharm Res. 2017;7(2):1-9.

6. Cai HD, Liu JN, Chen SJ, Cao G, Chen HJ. Research progress on chemical constituents, biological activity and clinical application of Houttuynia cordata. Chin Tradit Pat Med. 2019;41(11):2719-28.

7. Hosseinzadeh H, Nassiri-Asl M. Pharmacological Effects of Glycyrrhiza spp. and Its Bioactive Constituents: Update and Review. Phytother Res. 2015;29(12):1868-86.

8. Shingnaisui K, Dey T, Manna P, Kalita J. Therapeutic potentials of Houttuynia cordata Thunb. against inflammation and oxidative stress: A review. J Ethnopharmacol. 2018;220:35-43.

9. Pramod S, Geeta JnP, MSM R. Phytochemistry and Biological Activity Perspectives of Rheum Species. Nat Prod J. 2016;6(2):84-93.

10. Shang X, Pan H, Li M, Miao X, Ding H. Lonicera japonica Thunb.: ethnopharmacology, phytochemistry and pharmacology of an important traditional Chinese medicine. J Ethnopharmacol. 2011;138(1):1-21.

11. Zhang Q, Ye M. Chemical analysis of the Chinese herbal medicine Gan-Cao (licorice). J Chromatogr A. 2009;1216(11):1954-69.

12. Jiang M, Zhao S, Yang S, Lin X, He X, Wei X*, et al.* An "essential herbal medicine"-licorice: A review of phytochemicals and its effects in combination preparations. J Ethnopharmacol. 2020;249:112439.

13. Marchev A, Dinkova-Kostova A, György Z, Mirmazloum L, Aneva IY, Georgiev MI. Rhodiola rosea L.: from golden root to green cell factories. Phytochem Rev. 2016;15(4):515-36.

14. Verma RS, Padalia RC, Chauhan A, Singh VR. Chemical composition of leaves, inflorescence, whole aerial-parts and root essential oils of patchouli {Pogostemon cablin (Blanco) Benth.}. J Essent Oil Res. 2019;31(4):319-25.

15. van Beek T, Joulain D. The essential oil of patchouli,Pogostemon cablin: A review. Flavour Frag J. 2018;33(1):6-51.

16. Wang Z, Xia Q, Liu X, Liu W, Huang W, Mei X*, et al.* Phytochemistry, pharmacology, quality control and future research of Forsythia suspensa (Thunb.) Vahl: A review. J Ethnopharmacol. 2018;210:318-39.

17. Deng GT. Studies on Chemical Constituents of Dryopteris crassirhizoma Nakai. Guangdong Pharmaceutical University; 2015.

18. Gao ZP, Ali ZF, Zhao JP, Qiao L, Lei HM, Lu YR*, et al.* Phytochemical investigation of the rhizomes of Dryopteris crassirhizoma. Phytochem Lett. 2008;1(4):188-90.

19. Shen HL, Xiang NJ, Gan Q, Ni CM, Miao MM. Componental Analysis of Bitter Almond for Fatty Acid Contents by GC-MS. PTCA(Part B: Chem Anal). 2009;45(11):1302-7.

20. Ahn J, Chae HS, Chin YW, Kim J. Alkaloids from aerial parts of Houttuynia cordata and their anti-inflammatory activity. Bioorg Med Chem Lett. 2017;27(12):2807-11.

21. Shi QH, Zhu HL, Li KY. Composition of the Essential Oil from Bitter Almond. J Northwest Forestry Univ. 2003;18(3):73-5.

22. Li Y, Pham V, Bui M, Song L, Wu C, Walia A*, et al.* Rhodiola rosea L.: an herb with anti-stress, anti-aging, and immunostimulating properties for cancer chemoprevention. Curr Pharmacol Rep. 2017;3(6):384-95.

23. Panossian A, Wikman G, Sarris J. Rosenroot (Rhodiola rosea): traditional use, chemical composition, pharmacology and clinical efficacy. Phytomedicine. 2010;17(7):481-93.

24. Wiedenfeld H, Dumaa M, Malinowski M, Furmanowa M, Narantuya S. Phytochemical and analytical studies of extracts from Rhodiola rosea and Rhodiola quadrifida. Pharmazie. 2007;62(4):308-11.

25. Pu WL, Zhang MY, Bai RY, Sun LK, Li WH, Yu YL*, et al.* Anti-inflammatory effects of Rhodiola rosea L.: A review. Biomed Pharmacother. 2020;121:109552.

26. Jens R. Volatiles from rhizomes of Rhodiola rosea L. Phytochemistry. 2002;59(6):655-61.

27. LI KY, SHI QH, ZHU HL, TANG DR. Chemical Compositions in Bitter Almond. J Northwest Forestry Univ. 2004;19(2):124-6.
